# Proteomics of plasma-derived extracellular vesicles from human patients identifies biomarkers for monitoring visceral leishmaniasis therapy

**DOI:** 10.3389/fimmu.2025.1646335

**Published:** 2025-09-12

**Authors:** Ana Torres, Ana Montero-Calle, Marina Lozano-Rendal, Carmen Sánchez, Lorena Bernardo, Jose Carlos Solana, Juan Victor San Martin, Rodrigo Barderas, Javier Moreno, Eugenia Carrillo

**Affiliations:** ^1^ WHO Collaborating Centre for Leishmaniasis, Spanish National Center for Microbiology, Instituto de Salud Carlos III, Majadahonda, Spain; ^2^ Centro de Investigación Biomédica en Red de Enfermedades Infecciosas, Instituto de Salud Carlos III, Madrid, Spain; ^3^ Escuela de Doctorado, Universidad Autónoma de Madrid, Madrid, Spain; ^4^ Chronic Disease Program (UFIEC), Instituto de Salud Carlos III, Madrid, Spain; ^5^ Dept. of Infectious Diseases, Internal Medicine, Hospital Universitario de Fuenlabrada, Fuenlabrada/Madrid, Spain; ^6^ Centro de Investigación Biomédica en Red de Fragilidad y Envejecimiento Saludable (CIBERFES), Instituto de Salud Carlos III, Madrid, Spain

**Keywords:** extracellular vesicles, proteomics, plasma, biomarkers, visceral leishmaniasis, cured patients

## Abstract

**Introduction:**

The most severe form of leishmaniasis, visceral leishmaniasis (VL), lacks standardized validated early predictors of treatment success or relapse. To distinguish between active infection and successful treatment, we searched for protein biomarkers in plasma-derived extracellular vesicles (EVs).

**Methods:**

The proteomic profiles of EVs from immunocompetent patients with active VL (n=12) or 1, 3, or 6 months after completing a standard treatment regimen (n=12 each) were analyzed by LC-MS/MS. Six candidate biomarkers were further tested by ELISA in whole plasma.

**Results:**

132 human proteins were differentially expressed in active VL- versus successfully treated patients. Pathway analysis identified pathogenic mechanisms associated with VL and pathways related to effective cure. SAA is directly measurable in whole plasma and exhibits differential expression levels, emerging as a promising, easily measurable, non-specific prognostic biomarker for patient management. Remarkably, we also identified *Leishmania* spp. proteins in EV samples, indicating a new source of parasite biomarkers in human samples.

**Conclusion:**

Plasma EVs contain protein biomarkers that can be used to monitor the response to treatment, some of which are detectable in whole plasma after 1 month of treatment. Our study also provides a proteomic landscape of plasma EVs involved in VL, offering insight into the pathogenesis of this complex disease.

## 1 Introduction

Leishmaniasis is a vector-borne tropical disease caused by the protozoan parasite *Leishmania* spp. Among its clinical forms, visceral leishmaniasis (VL) is the most severe due to its high morbidity and mortality rates (1). Although VL treatment is usually effective, relapses are common, especially in immunocompromised patients (2–4), as *Leishmania* infection resolution largely depends on an efficient cell-mediated immune response (5, 6).

The current method of confirming final cure in patients with VL relies on WHO guidelines, which define this as the absence of clinical symptoms 6 months after completion of treatment (7). Although the diagnosis of VL can be effectively undertaken, no early predictors of cure have been standardized and validated. This happens specially in immunocompromised individuals, such prognostic biomarkers are crucial to reduce the likelihood of therapeutic relapse and improve patient health and welfare. Parasite levels in the blood decline rapidly after the first dose of treatment, limiting the utility of polymerase chain reaction (PCR) for this purpose, particularly in field settings where most clinical cases occur. Likewise, the detection of antibodies in serum against *Leishmania* antigens - such as those targeted by rK39 inmunochromatographic test (rK39-ICT) rapid test- often remain positive post-treatment, making them unsuitable for determining when cure has been achieved. Since resolution of leishmaniasis is mediated by a cellular immune response that activates host macrophages to eliminate the parasite, cellular biomarkers are considered the most promising tools for assessing treatment outcomes. While some prognostic biomarkers have been identified over the last few decades, none have been successfully translated to clinic practice for confirming cure during post treatment follow-up (8–10). A positive leishmanin skin test (LST) has been associated to protective immunity (11, 12), its use is being phased out due to its invasive nature and the lack of GMP-grade leishmanin (13). *Ex vivo* assays measuring lymphoproliferative responses (CPA) and interferon (IFN)-γ production, or cytokine release—such as IFN-γ, interleukin (IL)-2, tumor necrosis factor (TNF), IP10 (CXCL10), and MIG (CXCL9)—via whole-blood assay (WBA) have shown promise for confirming cure (10, 14); however, these approaches rely on non-commercial antigens which lack standardization and may yield inconsistent results. These limitations underscore the translational gap and the urgent need for new, field-adaptable biomarkers, ideally measurable in minimally processed samples.

Extracellular vesicles (EVs) are emerging as a promising source of biomarkers, particularly those derived from readily available samples like plasma. The potential of these EVs lies in their ability to provide valuable information on both physiological and pathological processes (15–17). Hence, by analyzing the proteome of EVs, specific proteins have been identified that are useful biomarkers for diagnosing several infectious diseases, monitoring disease progression, and early predicting treatment responses (18, 19).

The protein contents of EVs derived from blood samples from patients with a neglected disease have been recently explored (20–25). In the context of leishmaniasis, so far biomarker research has mostly focused on dogs (26, 27) or *in vitro* models (28–30). While informative, these studies have limitations in simulating the complexity of this disease.

There is therefore a need for proteomic studies in EVs from human VL samples to gain a deeper understanding of EV dynamics during the disease, and to identify early changes in their composition in response to treatment. These studies could help uncover the mechanisms through which *Leishmania* interacts with host cells, and how these interactions contribute to the progression of the disease and cure after treatment, including key immune processes like the recruitment of phagocytic cells to the infection site and the induction of pro-inflammatory cytokines such as IFN-γ and TNF, both essential for mounting a Th1 immune response. Indeed, research efforts such as these could help identify *Leishmania* proteins not yet described in plasma-derived EVs from human VL patients.

Despite extensive research, significant gaps remain in our understanding of persistence regarding the mechanisms that determine disease progression in *Leishmania*-infected individuals. In particular, it remains unclear why some individuals can control the infection asymptomatically, while others progress to clinical VL. This uncertainty is compounded by the complex triad of interactions between the parasite, the human host, and the sandfly vector, involving immune evasion strategies and host genetic factors that are not yet fully elucidated (31).

In this study, we compared the proteomic profiles of plasma EVs from immunocompetent patients with active visceral leishmaniasis caused by *L. infantum* before and after 1, 3, and 6 months of treatment with liposomal amphotericin B (LAmB). Our objective was to explore the underlying pathophysiological mechanisms associated with this disease, and to identify and validate early biomarkers of clinical remission and effective cure.

## 2 Materials and methods

### 2.1 Plasma samples

Plasma samples were obtained from whole blood (10 mL) collected in heparin tubes from immunocompetent adult patients from the same VL endemic area in Madrid between 2013 and 2017 and allowed to rest overnight at room temperature. Plasma was then transferred to clean tubes and stored at -80 °C until use, as part of the Collection for Leishmaniasis Research of the CNM (ISCIII), registered at the Spanish National Biobank Register under ref. number C.0000898 (Royal Decree Act 1716/2011, 18th November). The study protocol for patients returning for voluntary follow-up was approved by the Ethics Committee (APR 12–66 and APR 12-67) of the Hospital Universitario de Fuenlabrada, Madrid, Spain, and by the ISCIII Ethics Committee (CEI PI 78_2022).

Samples for EV biological and proteomics analyses corresponded to 46 patients: 12 diagnosed with active VL before receiving treatment, 11 patients 1 month after treatment completion (VL_Tx1M), 12 patients 3 months after treatment completion (VL_Tx3M), and 11 patients 6 months after treatment completion (VL_Tx6M). For ELISA analysis, samples from a further 38 patients were included at the same time points. There were similar distributions of gender and age across patient groups; detailed summary is provided in **Table 1**.

**Table 1:** Demographic and clinical characteristics of VL patient plasma samples included in the study, grouped by follow-up time points (1, 3, and 6 months posttreatment).

For Proteomics	VL (n=12)	VL_Tx1M (n=11)	VL_Tx3M (n=12)	VL_TX6M (n=11)
Gender (M/F)	9/3	4/7	7/5	6/5
Age (years)	43.08 ± 12.68	42.09 ± 10.66	43.08 ± 13.21	46.54 ± 14.74
Ethnicity (C/A/O)	6C/5A/1O	2C/8A/1O	3C/8A/1O	3C/8A
PCR (P/N)	11/1	0/11	0/12	0/11
rK39-ICT (P/N)	9/3	6/5	7/5	5/6
CPA/IFN-γ (P/N)	1/11	8/3	11/1	12/0
For ELISA	VL (n=10)	VL_Tx1M (n=9)	VL_Tx3M (n=10)	VL_Tx6M (n=8)
Gender (M/F)	8/2	3/6	5/5	3/5
Age (years)	44.1 ± 13.81	41.44 ± 11.80	45.20 ± 13.37	47.87 ± 17.17
Ethnicity (C/A/O)	4C/5A/1O	2C/7A	3C/6A/1O	2C/6A
PCR (P/N)	10/0	0/9	0/10	0/8
rK39-ICT (P/N)	6/4	5/4	6/4	4/4
CPA/IFN-γ (P/N)	1/9	6/3	10/0	8/0

M, male; F, female; C, Caucasian; A, African; O, Other; P, positive; N, negative.

Data are presented separately for the proteomics and ELISA subcohorts. Demographic data are presented as frequencies for ethnicity and gender, and age as mean ± standard deviation. Clinical data for PCR, rk39-ICT, and CPA/IFN-γ are indicated as numbers of positive (P) and negative (N) cases.

Active cases of VL were defined as patients with clinical symptoms and a confirmed diagnosis of leishmaniasis by the rK39-ICT and/or PCR (14).

Treated VL patients were defined as patients diagnosed with visceral leishmaniasis treated with the standard regimen of liposomal amphotericin B (3 mg/kg/day, days 1-5, 14 and 21) who showed no symptoms and tested PCR negative at 6-months after the end of treatment in accordance with WHO guidelines (32). Additionally, the cellular proliferation assay (CPA) and/or IFN-γ production to soluble *Leishmania* antigen (SLA) were considered as indicators of cure as described by Botana et al. (14). Patients enrolled in our study voluntarily agreed to attend follow-up appointments at the hospital 1-, 3- and 6-months post end-treatment to provide blood samples to look for prognostic biomarkers of cure.

Samples were randomly pooled according to predefined groups to create composite samples for TMT quantitative proteomics analysis using data dependent acquisition (DDA) (33). Pooling reduces the impact of individual variability and enhances the detection of specific protein biomarkers associated with disease or early cure (34, 35).

### 2.2 Isolation of EVs

Plasma samples from each patient cohort were thawed on ice and then pooled mixed equal volumes of plasma to give 2 mL per group. Pooled samples were first centrifuged at increasing speeds at 4°C (300 x g for 10 min; 2,000 x g for 30 min, and 12,000 x g for 30 min) and the supernatants diluted with equal volumes of filtered PBS 1x (Thermo Fisher Scientific, Waltham, MA, USA). Secondly, pooled samples were processed using a combined method of size exclusion chromatography and ultracentrifugation (SEC+UC) (36). In brief, samples were passed through a 70 nm/qEV SEC column (Izon Science, Christchurch, New Zealand), and fractions 6–9 of the flowthrough were collected. Next, these fractions were pelleted via two steps of ultracentrifugation (100,000 x g for 2 h 15 min at 4°C) in a Beckman Coulter Optima XPN-100 ultracentrifuge with a SW60Ti swinging-bucket rotor (Beckman Coulter Inc, CA, USA). The resulting pellet was resuspended in filtered PBS, and EVs were characterized by nanoparticle tracking analysis (NTA) and transmission electron microscopy (TEM) to assess their size, concentration, and intrinsic markers.

### 2.3 Nanoparticle tracking analysis

EV samples were diluted 1:50 in filtered PBS 1x and analyzed using a NanoSight NS300 instrument (Malvern, Worcestershire, UK), following previously validated settings (36). Briefly, serial dilutions were tested using healthy plasma-derived EVs to ensure particle concentration remained within the optimal detection range of the instrument. Two measurements per sample were made under consistent instrument settings, and data analysis was performed using NTA 3.2 Software. For each measurement, three videos were recorded, with the sample continuously infused through an automatic syringe pump at a flow rate of 50 μL/min. The parameters included: camera level 12, auto background subtraction/blur/minimum track, length acquisition time 60 s, 3 videos and detection threshold 5. Final concentration was expressed based on the dilution factor employed.

### 2.4 Protein quantification

EV samples were concentrated by lyophilization to ensure precise measurement of protein concentration for SDS-PAGE and proteomics studies. Subsequently, the samples were rehydrated with milliQ water to achieve a minimum concentration of 0.2 μg/μL for further analysis.

Protein concentration was measured using the Micro BCA Protein Assay Reagent Kit (Thermo Fisher Scientific, Waltham, MA, USA) following the manufacturer’s instructions. Briefly, a dilution of 1/20 of each sample was prepared in a final reaction of 200 μL of BCA Working Reagent. The reaction mixture was incubated for 2 h at 37°C. Absorbance was measured at 562 nm using a Multiskan FC (Thermo Fisher Scientific, MA, USA). Protein concentrations were calculated based on a standard curve prepared with bovine serum albumin (BSA) and fitted using a four-parameter logistic model.

### 2.5 Transmission electron microscopy

EV samples were examined by electron microscopy (TEM) through negative staining. Samples were diluted 1:10 in PBS1x and fixed in a final concentration of 2% paraformaldehyde for 5 min. Then, samples were placed on glow-discharged, carbon-coated copper grids for 5 min, following by two washes with MilliQ water. The samples were negatively stained with 2% aqueous uranyl acetate for 1 min. EV particles were visualized using a FEI Tecnai 12 electron microscope equipped with a LaB6 filament operating at 120 kV, and images were captured with an FEI Ceta digital camera at 30,000× magnification. Approximately 30 images were acquired per group to evaluate vesicle morphology and size distribution.

### 2.6 Statistical analysis of EV characterizations

Statistical analyses were conducted using the program GraphPad Prism version 9.0 (GraphPad Software Inc, CA, USA). Differences between groups were assessed by one-way analysis of variance (ANOVA) followed by Tukey’s honestly significant difference (HSD) *post hoc* test for multiple comparisons. Significance was set at three levels: *p < 0.05, **p < 0.01, and ***p < 0.001.

### 2.7 18-plex TMT labelling and peptide fractionation

For proteomics analysis, a TMT 18-plex experiment was performed with the plasma-derived EV samples, each of which was analyzed in triplicate. Digestion was performed with trypsin (1:20, trypsin:protein ratio) and SP3 magnetic beads, as previously described (33, 37, 38). Briefly, 10 µg of pooled EVs for each group were resuspended in RIPA buffer to a final volume of 100 µL and lysed by 5 cycles of 5 min of incubation on ice and 5 min of incubation at 95 °C. Proteins were then reduced, alkylated, and trypsin digested in a 100 μL solution of 200 mM HEPES at pH 8.0. The resultant supernatants containing the digested proteins were collected and TMT labelled. After labelling, peptides were separated into 12 fractions using a high pH reversed-phase peptide fractionation kit (Pierce, Thermo Fisher Scientific) in 0.1% triethylamine 2.5-100% ACN. Finally, fractions 1 and 12 were pooled together, and the 11 fractions were dried under vacuum and stored at -80 °C until analysis in an Orbitrap Exploris 480 mass spectrometer equipped with a FAIMS pro Duo interface (33).

### 2.8 LC–MS/MS analysis

Prior to LC-MS/MS, peptides were resuspended in 10 μL of 0.1% formic acid (FA) in mass spectrometry grade H_2_O and 4 μL of fractions 1 and 12, and 2 and 3, and 2 μL of fractions 4 to 11 (800 ng) were injected using the Vanquish Neo UHPLC System (Thermo Fisher Scientific, Waltham, MA, USA). For LC, samples were loaded into a precolumn PepMap 100 C18 3 µm, 75 µm × 2 cm Nanoviper Trap 1200BA (Thermo Fisher Scientific, Waltham, MA, USA) and eluted in an Easy-Spray PepMap RSLC C18 2 µm, 75 µm × 50 cm (Thermo Fisher Scientific, Waltham, MA, USA) heated at 50 °C, and eluted in a 120 min gradient using a flow rate of 300 nL/min and 0.1% FA H_2_O (buffer A) and 0.1% FA in 80% ACN (buffer B) as elution buffers. The 2 h gradient used was 0–2% buffer B for 4 min, 2% buffer B for 2 min, 2–42% buffer B for 100 min, 42–72% buffer B for 14 min, 72–95% buffer B for 5 min, and 95% buffer B for 10 min.

For MS/MS, standard parameters were used according to established protocols described previously enabling the turboTMT and FAIMS Pro Duo Interface (33, 39, 40). Briefly, a data dependent acquisition (DDA) method was used with 1900 V of liquid junction voltage and 280 °C capillary temperature used for ionization. The full scan was acquired with a m/z 350–1400 mass selection, an Orbitrap resolution of 60,000 (at m/z 200), an automatic gain control (AGC) value of 300%, and a maximum injection time (IT) of 25 ms. After the survey scan, the 12 most intense precursor ions were selected for MS/MS fragmentation, which was performed with a normalized collision energy of 34, and MS/MS scans were acquired with a 100 m/z first mass, an AGC target of 100%, a resolution of 15,000 (at m/z 200), an intensity threshold of 2 × 10^4^, an isolation window of 0.7 m/z units, a maximum IT of 22 ms, and the TurboTMT enabled. Charge state screening was enabled to reject unassigned, singly charged, and greater than or equal to seven protonated ions. A dynamic exclusion time of 30 s was used to discriminate against previously selected ions. For FAIMS, a gas flow of 4.7 L/min and CVs = −45 V and −60 V were used.

### 2.9 MS data and statistical analysis

MS data were analyzed with MaxQuant (version 2.4.2) using standardized workflows. Mass spectra *.raw files were searched against UniProt UP000005640_9606.fasta *Homo sapiens* (human) 2022 database (20,577 protein entries, March 2022) or against the *L. infantum* database (8,575 protein entries) using Reporter ion MS2 type. Combined searches against both databases were performed to minimize cross-species peptide assignment and increase the reliability of parasite protein identification. Standard MaxQuant parameters were used, and reporter ion intensities were bias corrected for overlapping isotope contributions from the TMT tags according to the manufacturer’s certificate (37–39). Raw proteomics data obtained with the Orbitrap Exploris 480 mass spectrometer equipped with FAINS pro DUO interface were deposited to the ProteomeXchange Consortium via the PRIDE partner repository with the dataset identifier PXD060604.

Next, data normalization, sample loading (SL) and Trimmed Mean of M-value (TMM) normalizations were carried out with R Studio (version 4.1.1) according to established protocols (https://github.com/pwilmart, accessed on 2 November 2022), using the “tidyverse”, “psych”, “gridExtra”, “scales”, and “ggplot2” packages (version 4.1.1). Finally, statistical analysis was performed using an empirical Bayes-moderated t-statistics method in R Studio (version 4.1.1) using the packages “limma”, “dplyr”, “tidyverse”, “ggplot2”, and “rstatix” according to previously described procedures (33, 37, 41).

### 2.10 Enzyme linked immunosorbent assay on whole plasma

Potential biomarkers identified though proteomics analysis were evaluated using commercial sandwich ELISA kits for plasma samples according to the manufacturer’s guidelines.

For each ELISA, 100 µL of both diluted plasma samples and standard curve solutions were added to pre-coated plates specific to each protein. The required dilution of each sample was selected based on the theoretical concentrations available in ‘The Human Protein Atlas’ database (https://www.proteinatlas.org/, accessed January 2, 2025) and confirmed through preliminary dilution test to ensure accurate detection within the dynamic range of the assay. The proteins assayed were: FN1 (plasma dilution 1:1,000; DY1918-05, Bio-Techne, MSP, USA), SAA1 (1:10; DY3019-05, Bio-Techne, MSP, USA), SLC4A1 (1:100; EH6632, FineTest, CO, USA), SERPINA1 (1:10,000; EH3968, FineTest, CO, USA), YWHAZ (1:10; CSB-EL026293HU, Cusabio, TX, USA), and ITGB1 (1:10; Orb563558, Biorbyt, Cambride, UK).

In brief, after incubation for 2 h at room temperature (RT), the plates were washed and biotin-conjugated detection antibody added for an additional 2 h at RT. After washing, streptavidin was added and incubated for 20 to 60 min based on the specific protein. The plates were later incubated with 3,3’,5,5’-tetramethylbenzidine (TMB) substrate solution, and absorbance measured at 450–540 nm using a Multiskan FC (Thermo Fisher Scientific, MA, USA). Data analysis was performed using a four-parameter logistic curve with GraphPad Prism software version 9.0 (GraphPad Software Inc, USA). To determine differences between samples, data were compared using a two-tailed Student’s t-test for normally distributed data. For data not showing a normal distribution, the Mann-Whitney U test was employed. Differences between groups were considered significant at p < 0.05.

### 2.11 Biological analysis

In all protein-level tests, proteins were considered significantly differentially expressed when showed a p-value ≤ 0.05 and a fold change (FC) ≥ 1.5 or ≤ 0.67. Functional enrichment analysis of the proteins quantified was performed using the Gene Ontology (GO) database through DAVID 2021 (42, 43). Protein-protein interactions were assessed using STRING (version 12.0) (44), while the most enriched pathways were identified using the Reactome database (version 3.7) (45). In all biological enrichment analyses, significance was evaluated using Fisher’s exact test, and Benjamini-Hochberg correction was applied to control the false discovery rate (FDR). Only terms or pathways with adjusted p-values (FDR) ≤ 0.01 were considered significant. A heatmap was generated to visualize differential protein expression in patient groups using Flaski toolbox (version 3.16.14) (46). Hierarchical clustering was applied to both samples and proteins based on expression similarity. Prior to clustering, data were normalized and transformed to z-scores (row-wise), allowing visualization of relative expression levels across conditions. The databases TritrypDB (VEuPathDB) (47) (https://tritrypdb.org/, accessed January 2, 2025), Wikidata (https://www.wikidata.org/, accessed January 2, 2025), and UniProt (48) (https://www.uniprot.org/, accessed January 2, 2025) were consulted to identify and characterize *Leishmania* proteins.

## 3 Results

### 3.1 Characterization and proteomics analysis of extracellular vesicles

**Figure 1:**
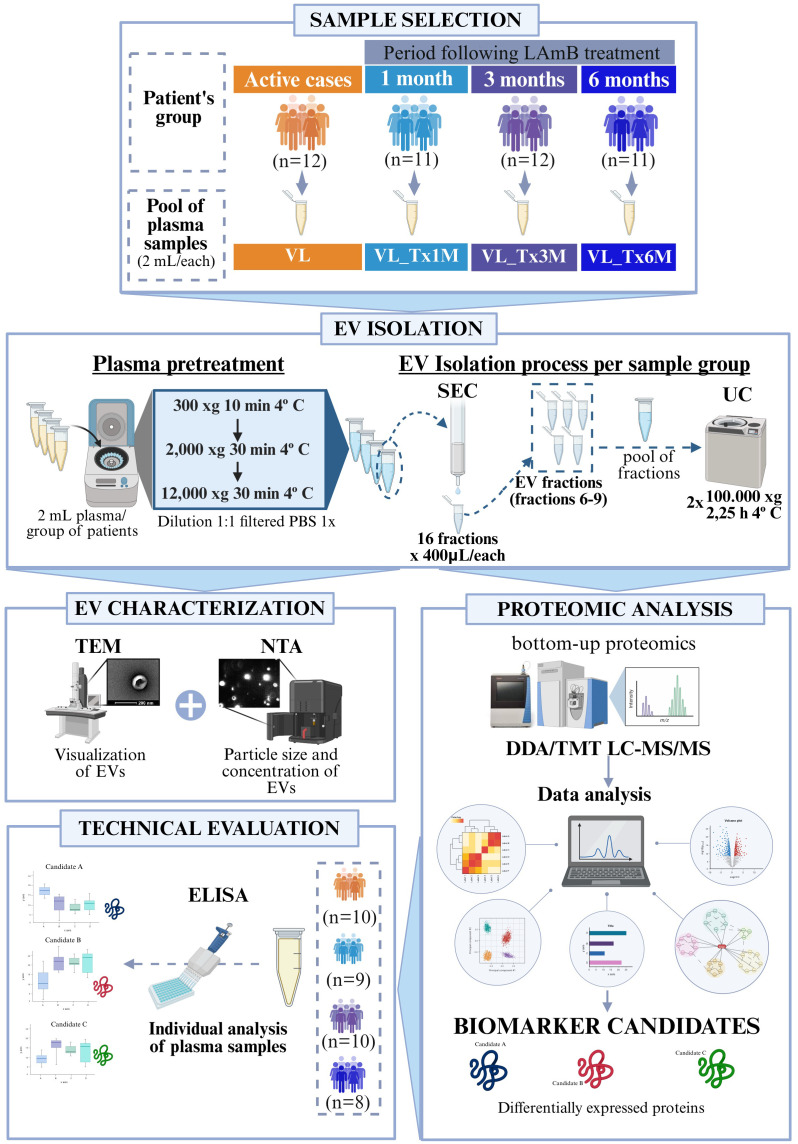
Workflow of the different techniques employed in this study. The flowchart illustrates the methodological steps: 1) Plasma samples from patients with active VL, or 1, 3, or 6 months after treatment; 2) EV isolation involving sample pretreatment followed by the combined SEC and UC method; 3) EV characterization using TEM, and NTA; 4) Bottom-up proteomic analysis using TMT LC-MS/MS followed by bioinformatics analysis to identify biomarker candidates; 5) Technical evaluation by ELISA assays on individual plasma samples to confirm the selected biomarkers.

Plasma EVs isolated from immunocompetent patients with VL and after 1, 3 or 6 months of treatment completion (VL_Tx1M, VL_Tx3M, and VL_Tx6M) were characterized according to different techniques as depicted in **Figure 1**.

**Figure 2:**
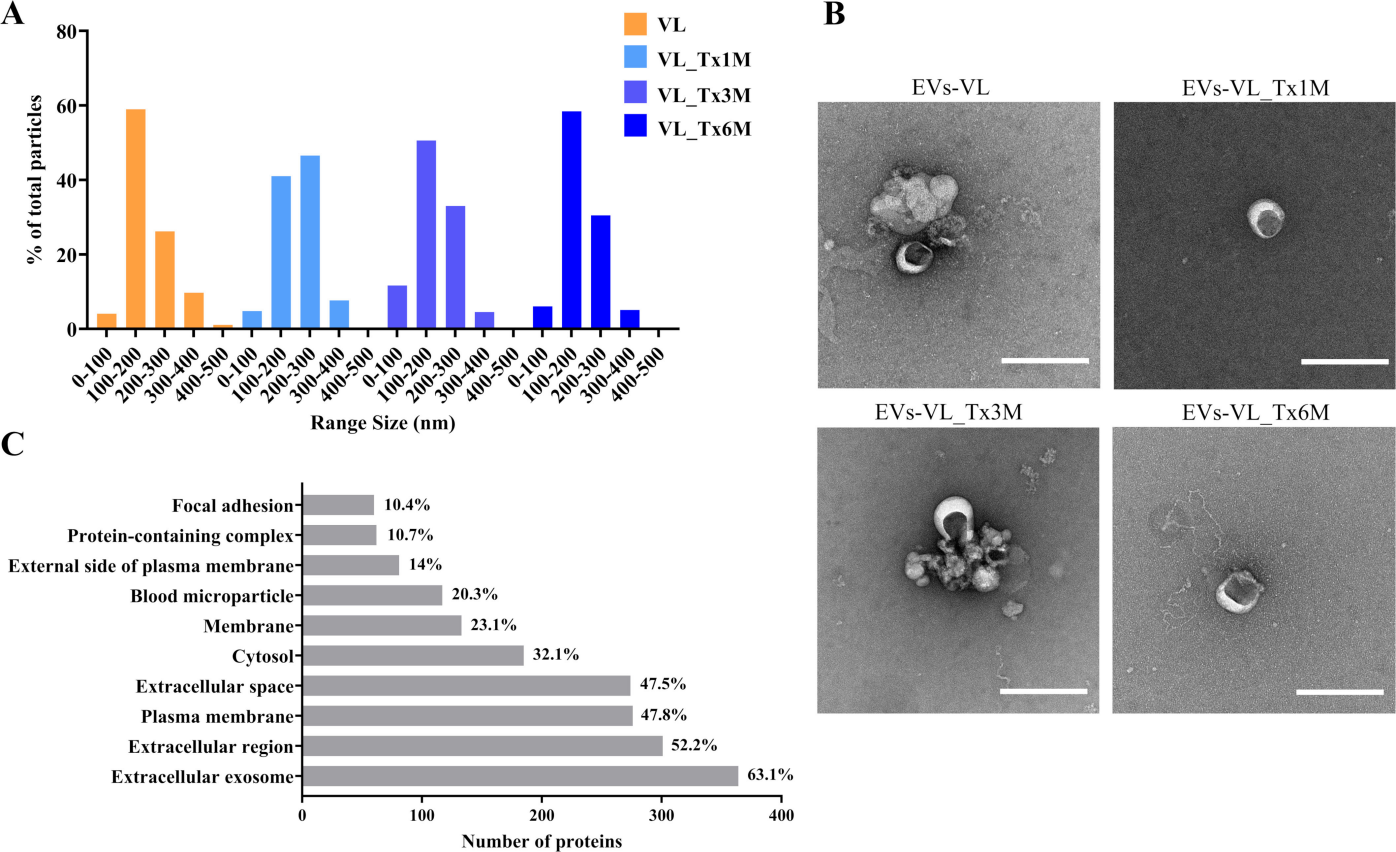
Characterization of EVs isolated and proteomics analysis. **(A)** Particle size distributions from NTA analysis of EVs from VL, VL_Tx1M, VL_Tx3M, and VL_Tx6M patients. Particle size distributions were expressed as percentages after normalization per total number of particles. **(B)** TEM images of the EVs (scale bar = 200 nm). **(C)** Top 10 enriched cellular component GO terms of EV proteins, with bars representing the number of proteins and percentages indicated.

NTA-determined particle sizes and distributions were consistent across all sample groups, indicating that the EVs isolated mainly contained particles within the 100–300 nm size range (**Figure 2A**). This range was confirmed by transmission electron microscopy (TEM) whereby the particles appeared cup-shaped alongside aggregates in all isolates (**Figure 2B**).

To assess protein integrity and potential contamination with abundant plasma proteins, SDS-PAGE was performed on EV samples from each patient group (**Supplementary Figure S1**). All preparations displayed well-defined protein banding patterns without signs of degradation. Albumin (~66 kDa) was detected in varying amounts across samples, though it was not a predominant component. Through proteomics analysis, we were able to identify 443 human proteins enriched in the EV samples. These proteins were quantified and analyzed for their differential expression, as well as subjected to bioinformatics analysis at a FDR ≤ 0.01 across all samples (**Supplementary Table 1**).

**Figure 3:**
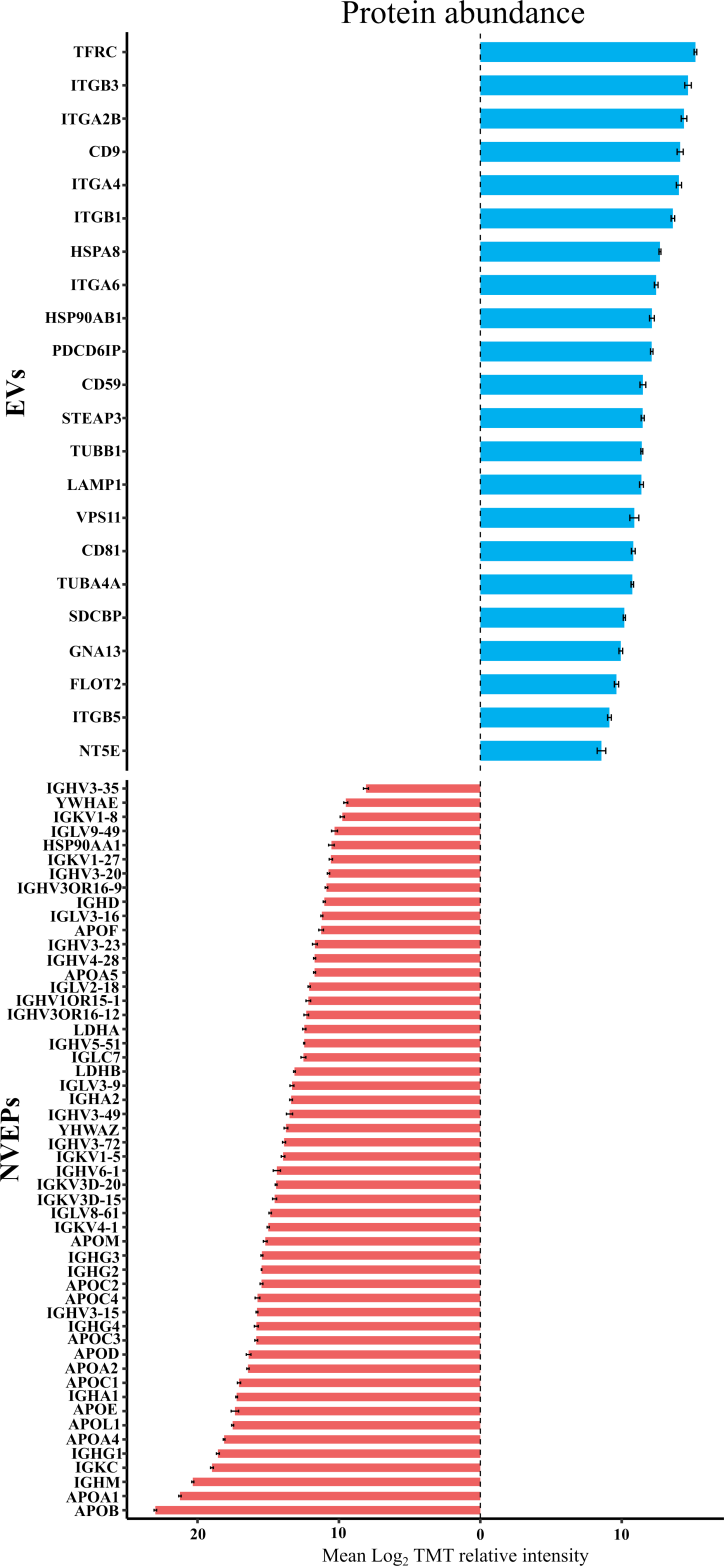
Relative abundance of selected proteins from plasma-derived extracellular vesicle (EV) and non-EV fractions (NVEPs). Barplot showing the mean relative intensity (log_2_TMT) of proteins classified according to MISEV2023 guidelines as EV-associated (blue bars) or non-EV proteins (red bars). Each bar corresponds to an individual protein, identified by its UniProt code. Protein classification was based on established EV markers, contaminants, and co-isolated components as defined in MISEV2023. The graph illustrates the relative abundance and distribution of representative proteins across both fractions, highlighting the enrichment or depletion of canonical EV proteins versus lipoproteins and plasma proteins frequently co-purified in EV preparations.

To check for the presence of EV-related proteins and potential contaminants in the samples, we conducted a detailed analysis in accordance with recommendations of Minimal information for studies on extracellular vesicles (MISEV2023) (49). Three categories were analyzed to confirm the presence of EVs as depicted in **Supplementary Table 2**. Almost all the protein markers of categories 1 (transmembrane or GPI-anchored proteins) and 2 (cytosolic proteins) were detected. Among the proteins identified for category 1 were tetraspanins CD81 and CD9, integrins (ITG), transferrin (TRF1), or 5’-nucleotidase (NT5E). In category 2, proteins such as flotillin (FLOT2), syntenin (SDCBP), heat shock proteins (HSPA8 and HSP90AB1), actins (ACTA and ACTB), or tubulins (TUBA and TUBB) were detected, among others. Plasma contaminant proteins (Category 3), such as immunoglobulins (IGH) and apolipoproteins (APO), were also detected in all samples. To further evaluate the enrichment of EV-associated proteins and the presence of co-isolated components, we compared the relative abundance (log_2_TMT intensity) of representative proteins from the three MISEV categories across EV and non-EV fractions (**Figure 3**). Most proteins enriched in the EV fraction corresponded to category 1b (single-pass transmembrane proteins), including canonical markers such as TRF1, ITGB3, and ITGA2B, as well as category 1a proteins (multipass transmembrane), such as CD9. These findings reinforce the vesicular origin of the isolated fractions. In contrast, proteins assigned to category 3 (potential plasma contaminants depicted as NVEP) were mainly enriched of apolipoproteins (APOA1, APOB) and immunoglobulins, such as IGHM and IGHK were particularly abundant. Their presence is consistent with the known co-isolation of soluble plasma proteins; however, this did not compromise the identification of *bona fide* EV markers. Altogether, the abundance profiles support the enrichment of vesicle-associated proteins in the EV fraction, despite the co-detection of plasma-derived components.

We then went on to analyze the GO enrichment of the cellular component (CC) category based on the DAVID database using the human genome as background. Out of 433 proteins, 364 showed strong association with extracellular vesicles, specifically with the enriched term ‘extracellular exosome’ (GO: 0070062), representing 63.1% of the identified proteins (**Figure 2C**). Moreover, other enriched terms such as ‘extracellular region’ (GO: 0005576), ‘plasma membrane’ (GO: 0005886), ‘extracellular space’ (GO: 0005615), and ‘cytosol’ (GO: 0005829) were notably prominent at percentages of 52.2%, 47.8%, 47.5%, and 32.1% respectively.

These results indicate that the isolated EVs in this study met the criteria for subsequent analysis.

### 3.2 Differences in the proteomic profiles of plasma-derived EVs in active VL- and successfully treated patients

**Figure 4:**
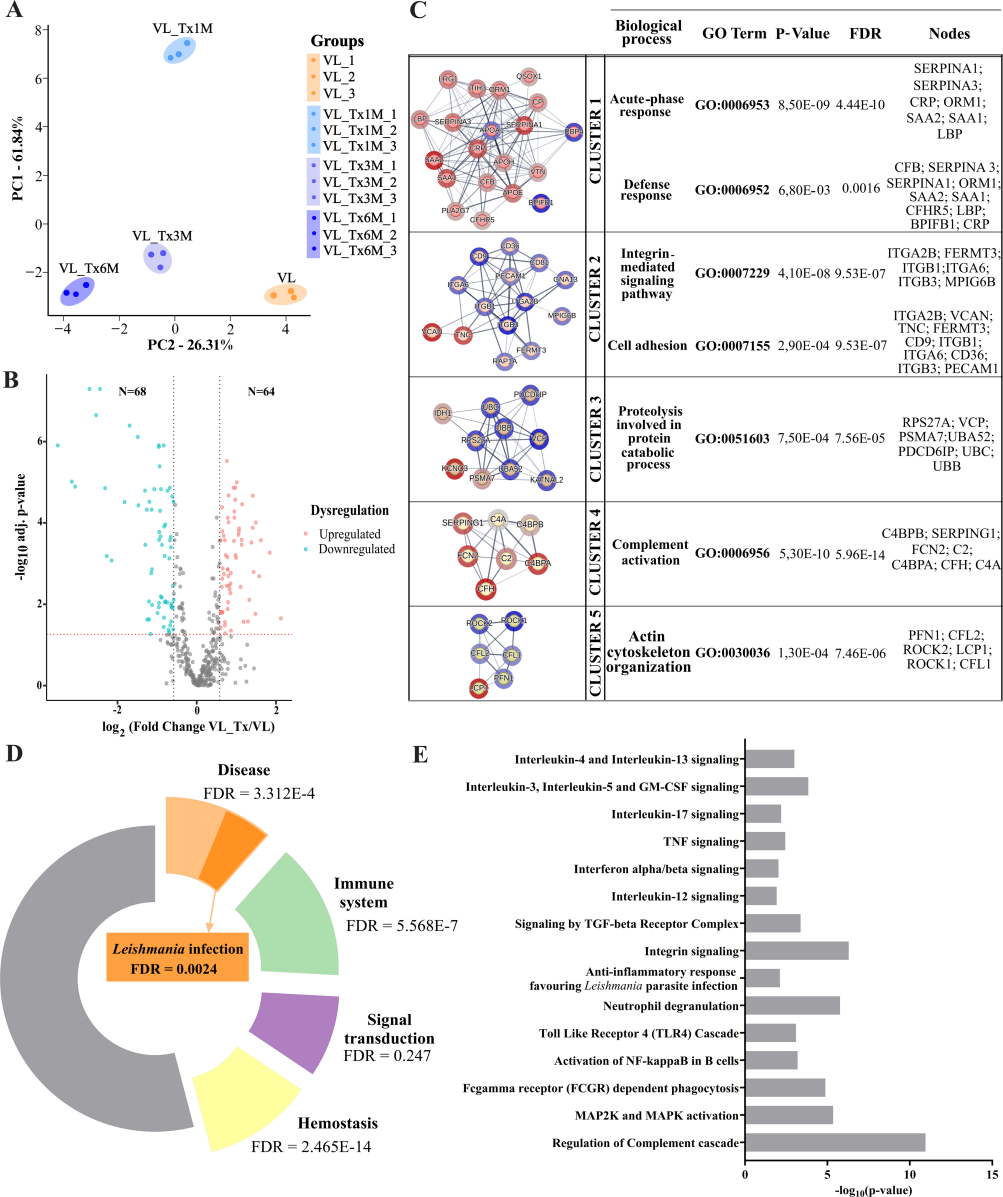
Proteomic profiles of VL and posttreatment groups-host proteins. **(A)** PCA plot showing variance distribution across different groups (VL, VL_Tx1M, VL_Tx3M, and VL_Tx6M) observed in triplicate samples. **(B)** Volcano plot illustrating differential protein expression levels in group VL versus VL_Tx. The two black dashed lines represent a log_2_fold change equal to 0.58 or -0.58. The red dashed line represents a FDR = 0.05. **(C)** Cluster analysis of dysregulated human proteins identified by STRING, highlighting the top five clusters associated with the *Leishmania* infection response. Upregulated proteins are marked in blue and downregulated proteins in red. Adjacent to each cluster network, associated biological processes, p-values, and FDR values, along with the proteins contributing to each process are indicated. **(D)** Donut chart of enriched pathways detected by Reactome. **(E)** Bar chart of enriched biological processes related to *Leishmania* infection and the immune response identified through Reactome analysis.

To determine whether active VL- and successfully treated patients could be distinguished based on their EV protein signatures, we performed a principal component analysis (PCA) including expression levels of the proteins identified. Four separate components, or clusters, corresponding to the four patient groups were detected indicating differences in protein expression profiles according to their disease state (**Figure 4A**).

In a volcano plot comparing protein expression levels in active VL- and successfully treated patients, all groups of treated patients merged (VL_Tx) separately to the active VL patients, or VL group. 132 differentially expressed proteins (DEPs) (FC ≥ 1.5, FC ≤ 0.67, p ≤ 0.05) were identified, 64 of which were upregulated and 68 downregulated in the treated patient compared to VL group (**Figure 4B**).

To gain insight into the biological roles of distinctively expressed EV proteins in active VL- and treated patients, we compared dysregulated proteins between the groups VL and VL_Tx through STRING analysis. This tool revealed intercellular interactions and different gene ontology categories (FDR = 0.01). A total of 20 clusters were identified and the top 5 clusters were further analyzed for the biological pathways of the genes involved in the modules (**Figure 4C**). Functions strongly associated with inflammatory processes were unveiled. Cluster 1 included defense response (10/1,394 proteins, p = 8.50E-09; FDR = 4.44E-10) and acute-phase response (7/42 proteins, p = 6.80E-03; FDR = 0.0016), both crucial for activating the immune system during infection. Cluster 2 involved integrin-mediated signaling (6/100 proteins, p = 4.10E-08; FDR = 9.53E-07) and cell adhesion (10/965 proteins, p = 2.90E-04; FDR = 9.53E-07), emphasizing cell communication and attachment. Cluster 3 was enriched in proteolysis involved in protein catabolic processes (7/649 proteins, p = 7.50E-04; FDR = 7.56E-05), reflecting protein degradation processes. Cluster 4 focused on complement activation (7/60 proteins, p = 5.30E-10; FDR = 5.96E-14), a key component of innate immunity, and Cluster 5 on actin cytoskeleton organization (6/547 proteins, p = 1.30E-04; FDR = 7.46E-06), which is important for cell structure and movement. The upregulated and downregulated proteins in each cluster are listed in columns alongside the corresponding cluster (**Figure 4C**).

Additionally, a Reactome pathway analysis was conducted to characterize the main pathways associated with the significantly altered proteome in patients with active disease compared to treated patients, to further support the involvement of inflammation-related pathways identified in the STRING clusters. Among the pathways identified, several key processes were enriched in the majority of these DEP, including signal transduction (30/2,606 proteins, FDR = 2.47E-1), hemostasis (40/727 proteins, FDR = 2.46E-14), immune system (50/2,219 proteins, FDR = 5.57E-7), and disease pathways (40/2,188 proteins, FDR = 3.31E-4), as shown in **Figure 4D**. Notably, within the disease group, the *Leishmania* infection pathway was particularly enriched in DEP (9/254 proteins, FDR = 2.39E-3).

In a more in-depth analysis of the most enriched groups, we identified several key processes associated with the immune response to *Leishmania*. These included signaling pathways mediated by interleukin-17 (4 proteins, p = 0.006; FDR = 0.006), interferon alpha/beta (4 proteins, p = 0.009; FDR = 0.009), TNF (4 proteins, p = 0.003; FDR = 0.004), interleukin-12 (3 proteins, p = 0.01; FDR = 0.01), interleukin-3, interleukin-5 and GM-CSF (5 proteins, p = 1.42E-4; FDR = 5.69E-4). We also observed pathways involving receptor activation, including the ‘toll-like receptor 4 (TLR4) cascade’ (7 proteins, p = 8.02E-4; FDR = 0.001) and ‘FCGR-dependent phagocytosis’ (10 proteins, p = 1.32E-5; FDR = 1.59E-4). Additionally, pathways related to communication processes, such as ‘integrin signaling’ (6 proteins, p = 5.10E-7; FDR = 1.88E-5), and host defense mechanisms like ‘neutrophil degranulation’ (18 proteins, p = 1.71E-6; FDR = 5.07E-5) and ‘regulation of the complement cascade’ (15 proteins, p = 1.11E-11; FDR = 1.39E-9) were enriched. Further, cascade-like pathways, such as ‘MAP2K and MAPK activation’ (6 proteins, p = 4.52E-6; FDR = 8.13E-5), were also identified. Other pathways highlighted were related to anti-inflammatory responses, including the pathway ‘anti-inflammatory responses that favor *Leishmania* parasite infection’ (6 proteins, p = 0.007; FDR = 0.007) and ‘interleukin-4 and interleukin-13 signaling’ (6 proteins, p = 0.001; FDR = 0.002) (**Figure 4E**). The full list of pathways identified through Reactome, along with the corresponding proteins involved in each pathway, is provided in **Supplementary Table 3**.

### 3.3 Differential abundance of EV proteins in VL- and treated patients as potential host biomarkers of cure

**Figure 5:**
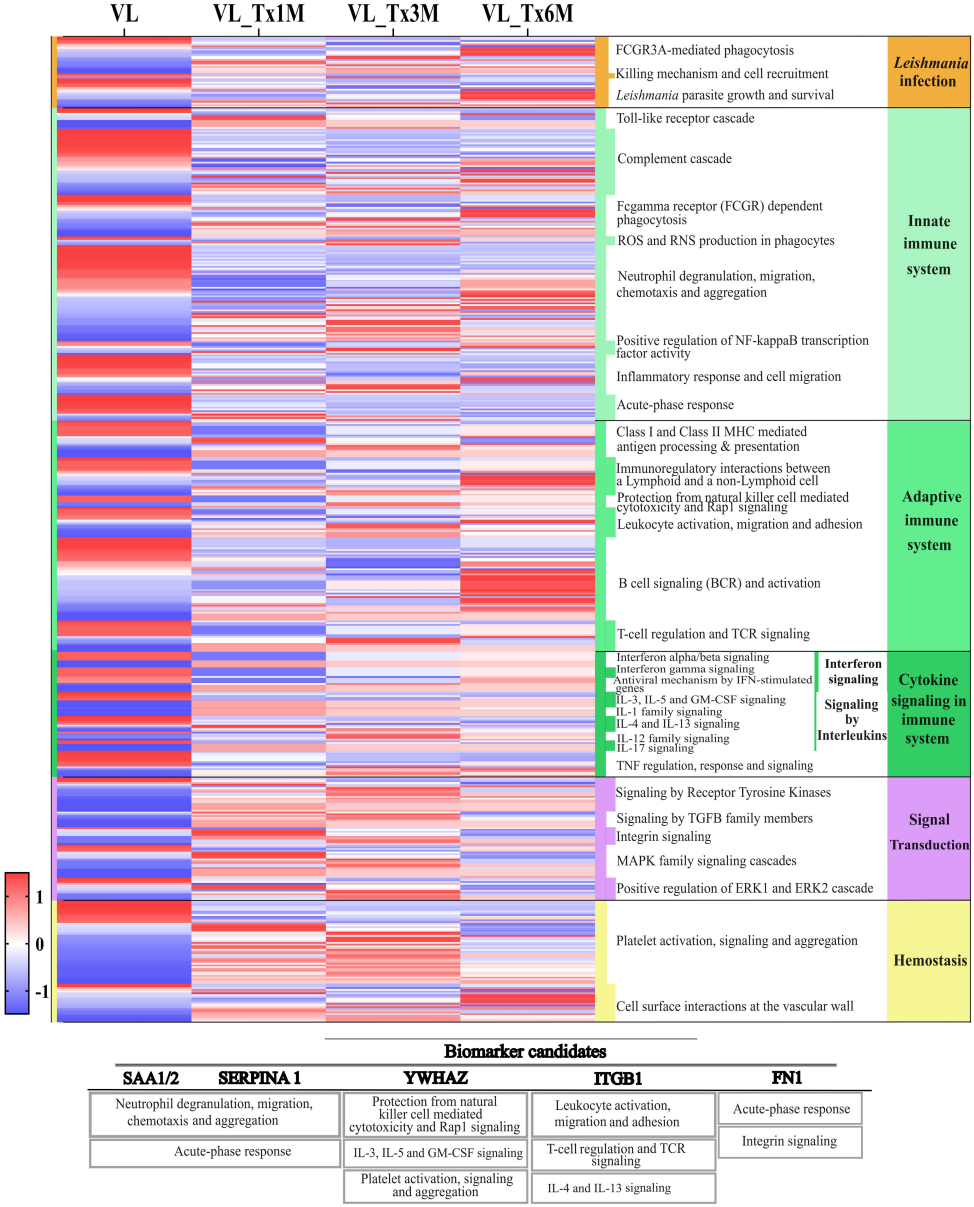
Heatmap of the proteomic profiles of the patient groups VL and VL_Tx1M, VL_Tx3M, and VL_Tx6M across different pathways. Columns represent the human proteins identified in each group, and rows indicate expression levels of these proteins across the groups. Protein expression levels were normalized using z-score transformation and visualized on a red-to-blue scale indicating relative enrichment or depletion, respectively. Associated Reactome pathway categories are shown on the right side of the heatmap. Additionally, a table with the biomarker candidates is included below, indicating each candidate protein and its principal associated biological processes.

Our volcano plot analysis was designed to examine the proteomic signatures of patients at different time points after treatment and to compare these to that of patients with active disease. Differences were detected among groups in the total number of DEPs, with 178 identified in the 1- and 3- month posttreatment groups, and 153 in the 6-month posttreatment group, suggesting that treatment affects the overall protein signature of patients (**Supplementary Figure 2**). To further investigate these temporal changes in proteomic profiles during treatment, a heatmap was prepared with expression values of all quantifiable proteins. This analysis was based on described categories, with detailed pathways from the DAVID and Reactome databases included for a more comprehensive assessment. Our results illustrated in **Figure 5** highlight not only the most enriched pathways related to the immunology of *Leishmania* infection, but also revealed distinct clusters of proteins with unique temporal expression patterns (**Supplementary Table 4**).

Distinct expression patterns were observed in pathways associated with *Leishmania* infection in patients with VL compared to treated patients. In samples from patients with active VL, ‘FCGR3A-mediated phagocytosis’ and ‘*Leishmania* parasite growth and survival’ were pathways showing most of their proteins downregulated (**Figure 5**). From the first month posttreatment, a change in DEPs was observed, until 6 months, when a contrasting profile to that of active VL was observed in that 11/15 and 9/14 proteins were upregulated, respectively for the two pathways. Further, proteins linked to the ‘killing mechanisms and cell recruitment’ pathway were upregulated during active VL, whereas 2/3 of these proteins were negatively expressed in the 3- and 6-month posttreatment groups (**Figure 5**).

The innate immune system pathways ‘complement cascade’, ‘inflammatory response and cell migration’ and ‘acute-phase response’ were highly enriched in the active VL group. As with the *Leishmania* infection pathways described above, the proteins involved in these three pathways were significantly downregulated in the 1- and 3-month posttreatment groups. Particularly, for the ‘acute-phase response’, 10 DEPs were all downregulated in VL_Tx6M (**Supplementary Table 4**). In relation to the proteins involved in the ‘neutrophil degranulation, migration, chemotaxis, and aggregation’ pathway, we observed a mixed expression profile with half of the 55 identified proteins enriched and the other half depleted during VL. 70% (25/36) of the upregulated proteins in this pathway were underexpressed in VL_Tx1M. However, this depleted expression pattern was slightly modified in the other treatment groups compared to the active VL group (54% and 53% of proteins downregulated in VL_Tx3M and VL_Tx6M, respectively).

Within the adaptive immune response, the most obvious change in protein expression was noticed in the ‘B cell signaling (BCR) and activation’ pathway, for which 71% of dysregulated proteins in the VL group (22/31) were upregulated in VL_Tx6M. However, in the earlier stages of treatment, this pathway showed no significant differences in DEPs. In the pathways ‘protection from natural killer cells mediated cytotoxicity and Rap1 signaling’ and ‘leukocyte activation, migration, and adhesion’, 3/3 and 8/11 proteins were upregulated in VL_Tx3M and VL, respectively, and this situation persisted 6 months after treatment. Among proteins related to ‘T-cell regulation and TCR signaling’, 8/14 (57%) were still downregulated 1 month after treatment but this scenario was reversed at VL_Tx3M and VL_Tx6M, when most proteins were significantly upregulated compared to the VL group.

Within the ‘interferon signaling’ pathways, in which we found proteins involved in ‘interferon alpha/beta signaling’ and ‘interferon gamma signaling’, consistent significant dysregulation was observed after the first month of treatment. This group was the only one in which downregulated proteins versus the VL group were identified (13/23, 57%). When we considered pathways related to interleukin signaling, we found that 50%-56% proteins (17/34 at VL_Tx1M and VL_Tx6M, 19/34 at VL_Tx3M) were significantly upregulated in the VL_Tx groups compared to VL. Particularly ‘IL-3, IL-5, and GM-CSF signaling’ and ‘IL-17 signaling’ pathways showed sustained upregulation over the first month posttreatment.

Finally, in signal transduction and hemostasis-related pathways, proteins were mostly downregulated in active VL. Following treatment, significant enrichment was observed compared to VL, particularly 1 and 3 months after treatment, suggesting the recovery of these processes. This trend was particularly evident in pathways like ‘signaling by TGF-beta family members’, ‘MAPK family signaling cascade’, and ‘platelet activation, signaling, and aggregation’.

**Figure 6:**
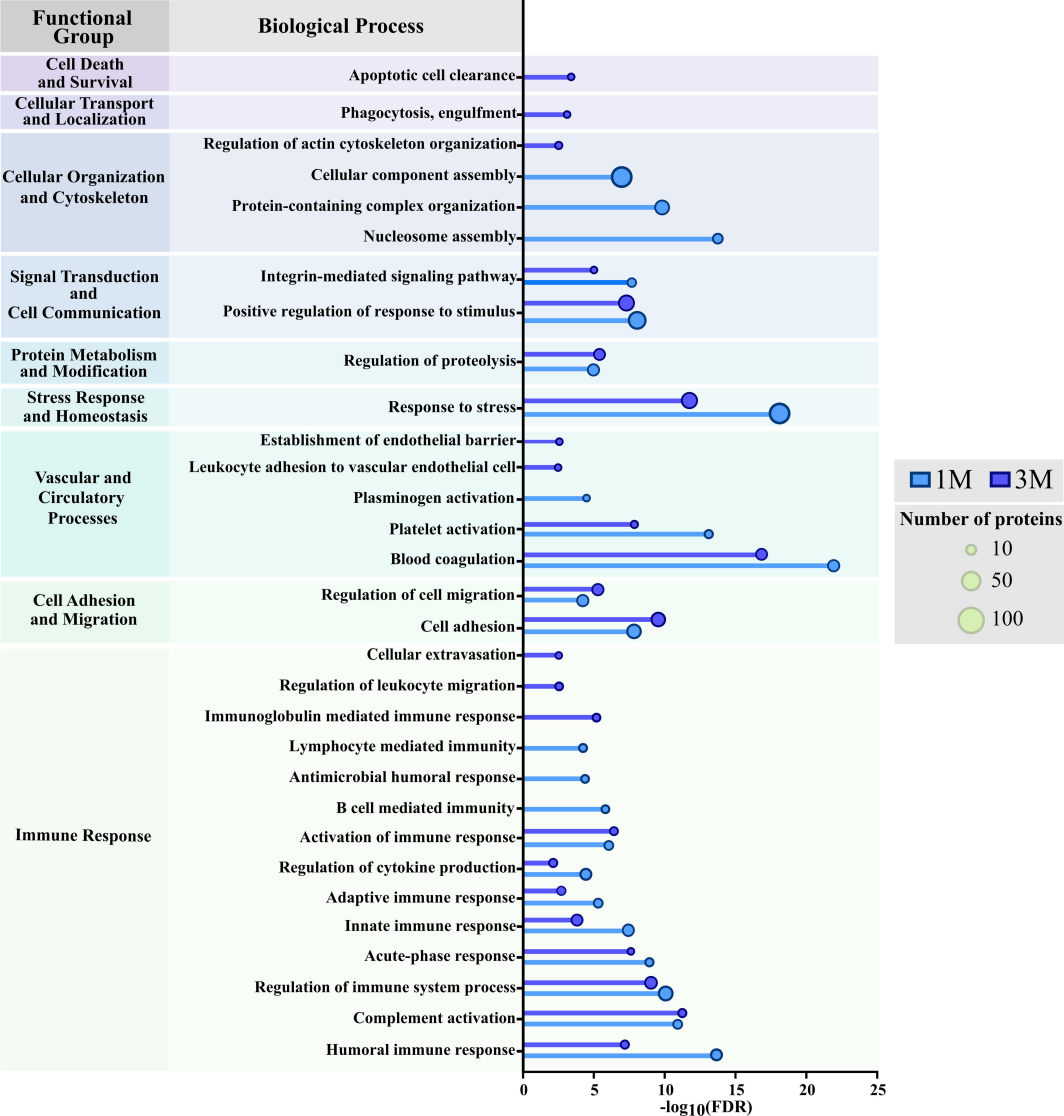
GO analysis of biological processes associated with proteins dysregulated in patients 1 month (1M) or 3 months (3M) posttreatment. The bar chart illustrates the most significantly enriched biological processes (FDR ≤ 0.01) grouped into functional categories. Bars indicate the significance of each process (-log10(FDR)), with lighter blue bars representing 1M processes and darker blue bars representing 3M processes. Circle sizes (small, medium, large) indicate the number of proteins contributing to each process, as shown in the key. Processes are classified into functional groups.

These results offer information on the specific protein signature associated with plasma-derived EVs from individuals affected by this disease, including differences in its expression produced in response to treatment. To further explore the biological significance of early posttreatment changes, we performed two separate Gene Ontology (GO) enrichment analyses on proteins found dysregulated 1 month and 3 months after treatment, in an effort to identify the most affected biological processes during the initial stages of posttreatment recovery (**Supplementary Table 5**). In total, 405 biological processes were identified, with 208 shared between both time points, 116 unique to VL_Tx1M and 81 to VL_Tx3M. The most enriched biological terms, including those common to both treatment time points and those unique to each, are illustrated in **Figure 6**, with processes grouped by functional category.

Common processes between the 1- and 3-month posttreatment time points suggest an ongoing process of immune response modulation compared to VL. Among them, ‘humoral immune response’ (FDR = 4.51E-14 and 1.33E-07), ‘innate immune response’ (FDR = 9.85E-08 and 3.8E-04), ‘adaptive immune response’ (FDR = 9.38E-06 and 3.4E-03), and ‘regulation of cytokine production’ (FDR = 9.17E-05 and 0.014) were highly enriched in VL_Tx1M compared to VL_Tx3M. ‘B cell mediated immunity’, ‘antimicrobial humoral response’ and ‘lymphocyte mediated immunity’ were only enriched after 1 month of treatment, whereas ‘immunoglobulin mediated immune response’, ‘regulation of leukocyte migration’, and ‘cellular extravasation’ were only present after 3 months. In addition, other common processes with higher quantities of proteins involved at 1 month were ‘response to stress’ (FDR = 3.82E-18 and 4.94E-12), ‘integrin-mediated signaling pathways’ (FDR = 4.51E-08 and 1.55E-05) or ‘platelet activation’ (FDR = 1.60E-13 and 2.65E-08). In contrast, among the common processes found to be more enriched at 3 months we identified ‘cell adhesion’ (FDR = 3.87E-08 at 1M vs 5.05E-10 at 3M) and ‘regulation of cell migration’ (FDR = 0.00013 and 1.29E-05). Finally, the processes ‘apoptotic cell clearance’ or ‘phagocytosis, engulfment’ were only identified at 3 months posttreatment.

Potential biomarker candidates were then identified by considering fold change values (FC ≥ 1.5, FC ≤ 0.67) of proteins involved in several of the described pathways using data from **Supplementary Table 1**. We focused on proteins found to be dysregulated from the first to the third month after treatment, which are considered early stages of cure (**Table 2**). Further, we selected proteins whose biological processes and associated pathways were related to immune response and parasite infection, as these are critical for VL progression and treatment response. Among proteins showing different expression patterns over time, the heat shock protein HSP90AB1, alpha-1-antitrypsin (SERPINA1), serum amyloid A-1 and A-2 (SAA-1; SAA-2) and plastin-2 (LCP1) were consistently downregulated in all three treatment groups. SERPINA1 and SAA-1/SAA-2 showed the lowest expression of all proteins, FC VL_Tx1M = 0.14 (p = 2.71E-11; FDR = 6.01E-9) and FC VL_Tx3M = 0.18 (p = 1.01E-7; FDR = 2.89E-6), respectively, and were therefore considered for further validation analysis. Both these proteins play a role in ‘neutrophil degranulation, migration, chemotaxis and aggregation’ and ‘acute-phase response’ pathways. Other downregulated proteins related to both the innate and adaptive immune system pathways, were S100-A9 (FC = 0.61; p = 9.85E-5; FDR = 0.0003) and CAMP (FC = 0.28; p = 4.31E-9; FDR = 1.12E-7), which were only found significantly dysregulated 1 month after treatment compared to the state of active disease. In addition, kiminogen-1 (KNG1) and vascular cell adhesion protein 1 (VCAM1), involved in ‘inflammatory response and cell migration’, were only dysregulated at VL_Tx3M (FC = 0.63, p = 9.15E-6 and FDR = 6.64E-5 and 0.42, p = 0.0001 and FDR = 0.0006, respectively).

**Table 2:** Candidate EV protein biomarkers based on their roles in pathways related to the immune response and *Leishmania* infection comparing samples from treated patients to those with VL.

Protein ID	Protein	Gene	VL_Tx1M (FC; p-value; FDR)	VL_Tx3M (FC; p-value; FDR)	VL_Tx6M (FC; p-value; FDR)	VL_Tx (FC; p-value; FDR)
P02730	Band 3 anion transport protein	SLC4A1	Upregulated (8.09; p=1.24E-10; FDR = 1.37E-08)	Upregulated (3.44; p=1.29E-7; FDR = 3.30E-6)	Upregulated (1.54; p=0.01; FDR = 0.02)	Upregulated (4.35; p=0.007; FDR = 0.02)
P05556	Integrin beta-1	ITGB1	Upregulated (1.92; p=7.87E-7; FDR= 5.81E-6)	Upregulated (2.08; p= 1.90E-6; FDR = 2.29E-5)	Upregulated (1.70; p=1.77E-5; FDR = 0.0001)	Upregulated (1.90; p=4.75E-7; FDR = 1.36E-5)
P63104	14-3–3 protein zeta/delta	YWHAZ	Upregulated (2.12; p= 7.60E-8; FDR= 1.02E-6)	Upregulated (2.06; p=2.09E-6; FDR=2.31E-5)	Upregulated (1.69; p=2.62E-5; FDR=0.0001)	Upregulated (1.96; p=1.58E-6; FDR=2.69E-5)
P35579	Myosin-9	MYH9	Upregulated (1.68; p=1.54E-6; FDR=9.77E-6)	Upregulated (2.57; p=2.71E-8; FDR=1.50E-6)	Upregulated (1.74; p=5.72E-7; FDR=8.45E-6)	Upregulated (1.98; p=0.0001; FDR=0.0007)
Q6UX06	Olfactomedin-4	OLFM4	Upregulated (1.78; p=0.0008; FDR=0.001)	Upregulated (2.72; p=3.25-5; FDR=0.0001)	Upregulated (2.07; p=0.0002; FDR=0.0008)	Upregulated (2.19; p=5.05E-5; FDR=0.0003)
P21926	CD9 antigen	CD9	Upregulated (1.93; p=1.65E-7; FDR=1.87E-6)	Upregulated (4.03; p=9.63E-8; FDR=2.89E-6)	Upregulated (2.14; p=6.42E-7; FDR=9.18E-6)	Upregulated (2.70; p=0.0003; FDR=0.001)
P02751	Fibronectin	FN1	Upregulated (2.44; p=1.56E-9; FDR=5.77E-8)	–	–	–
P01009	Alpha-1-antitrypsin	SERPINA1	Downregulated (0.14; p=2.71E-11; FDR=6.01E-9)	Downregulated (0.17; p=2.25E-7; FDR=4.98E-6)	Downregulated (0.13; p=9.68E-9; FDR=8.14E-7)	Downregulated (0.15; p=2.30E-10; FDR=5.10E-8)
P0DJI8	Serum amyloid A-1 protein	SAA1	Downregulated (0.20; p=3.86E-9; FDR=1.07E-7)	Downregulated (0.18; p=1.01E-7; FDR=2.89E-6)	Downregulated (0.16; p=2.42E-8; FDR=1.34E-6)	Downregulated (0.18; p=2.21E-10; FDR=5.10E-8)
P0DJI9	Serum amyloid A-2 protein	SAA2	Downregulated (0.12; p=5.83E-11; FDR=8.61E-9)	Downregulated (0.07; p=1.06E-8; FDR=6.73E-7)	Downregulated (0.05; p=1.96E-11; FDR=8.67E-9)	Downregulated (0.08; p=2.24E-8; FDR=1.24E-6)
P08238	Heat shock protein HSP 90-beta	HSP90AB1	Downregulated (0.41; p=7.07E-7; FDR=5.49E-6)	Downregulated (0.37; p=7.96E-7; FDR=1.26E-5)	Downregulated (0.51; p=8.02E-5; FDR=0.0003)	Downregulated (0.43; p=1.85E-6; FDR=3.01E-5)
P13796	Plastin-2	LCP1	Downregulated (0.36; p=9.66E-7; FDR=6.79E-6)	Downregulated (0.37; p=4.06E-7; FDR=8.17E-6)	Downregulated (0.63; p=0.0009; FDR=0.002)	Downregulated (0.45; p=0.0002; FDR=0.001)
P01042	Kininogen-1	KNG1	–	Downregulated (0.63; p=9.15E-6; FDR=6.64E-5)	–	–
P19320	Vascular cell adhesion protein 1	VCAM1	–	Downregulated (0.42; p=0.0001; FDR=0.0006)	–	–
P06702	Protein S100-A9	S100A9	Downregulated (0.61; p=9.85E-5; FDR=0.0003)	–	–	–
P49913	Cathelicidin antimicrobial peptide	CAMP	Downregulated (0.28; p=4.31E-9; FDR=1.12E-7)	–	–	–

Indicated for each protein, are the UniProt Protein ID, protein name, and gene name. The dysregulation of the different protein biomarkers is indicated for each posttreatment time point (VL_Tx1M; VL_Tx3M and VL_Tx6M) and across all treated patient groups (VL_Tx), along with their corresponding fold change (FC), p-values and FDR. Dashes denote no dysregulation observed.

In contrast, myosin-9 (MYH9), olfactomedin-4 (OLFM4), 14-3–3 protein zeta/delta (YWHAZ), CD9 and integrin beta-1 (ITGB1) were upregulated across all treatment groups. YWHAZ emerged as a possible candidate due to its consistent upregulation in the early posttreatment stages compared to VL (FC VL_Tx1M = 2.12, p = 7.60E-8, FDR = 1.02E-6; VL_Tx3M = 2.06, p = 2.09E-6, FDR = 2.31E-5). This protein participates in ‘IL-3, IL-5, and GM-CSF signaling’, ‘platelet activation, signaling and aggregation’ and ‘protection from natural killer cell mediated cytotoxicity’ pathways. ITGB1 was found to be predominantly upregulated 3 months after treatment (FC VL_Tx3M = 2.08, p = 1.90E-6, FDR = 2.29E-5). This protein plays a role in ‘leukocyte activation, migration and adhesion’, ‘T-cell regulation and TCR signaling’ and ‘IL-4 and IL-13 signaling’. Finally, fibronectin (FN1), which is involved in ‘acute-phase response’, ‘IL-4 and IL-13 signaling’ and ‘integrin signaling’, was only found upregulated at 1 month. This protein showed a FC = 2.44 (p = 1.56E-9; FDR = 5.77E-8) compared to the VL state.

The band 3 anion transport protein (SLC4A1) emerged as another protein of interest due to its remarkable increase in expression noted early after treatment, with its highest fold change observed at 1 month posttreatment (FC VL_Tx1M = 8.09, p = 1.24E-10, FDR = 1.37E-8).

### 3.4 Potential biomarkers for monitoring VL treatment

To identify host proteins relevant to *Leishmania* infection in whole plasma by ELISA, we searched for those proteins showing fold change differences in the VL and early posttreatment groups by examining SAA1/2, SERPINA1, SLC4A1, ITGB1, FN1, and YWHAZ.

**Figure 7:**
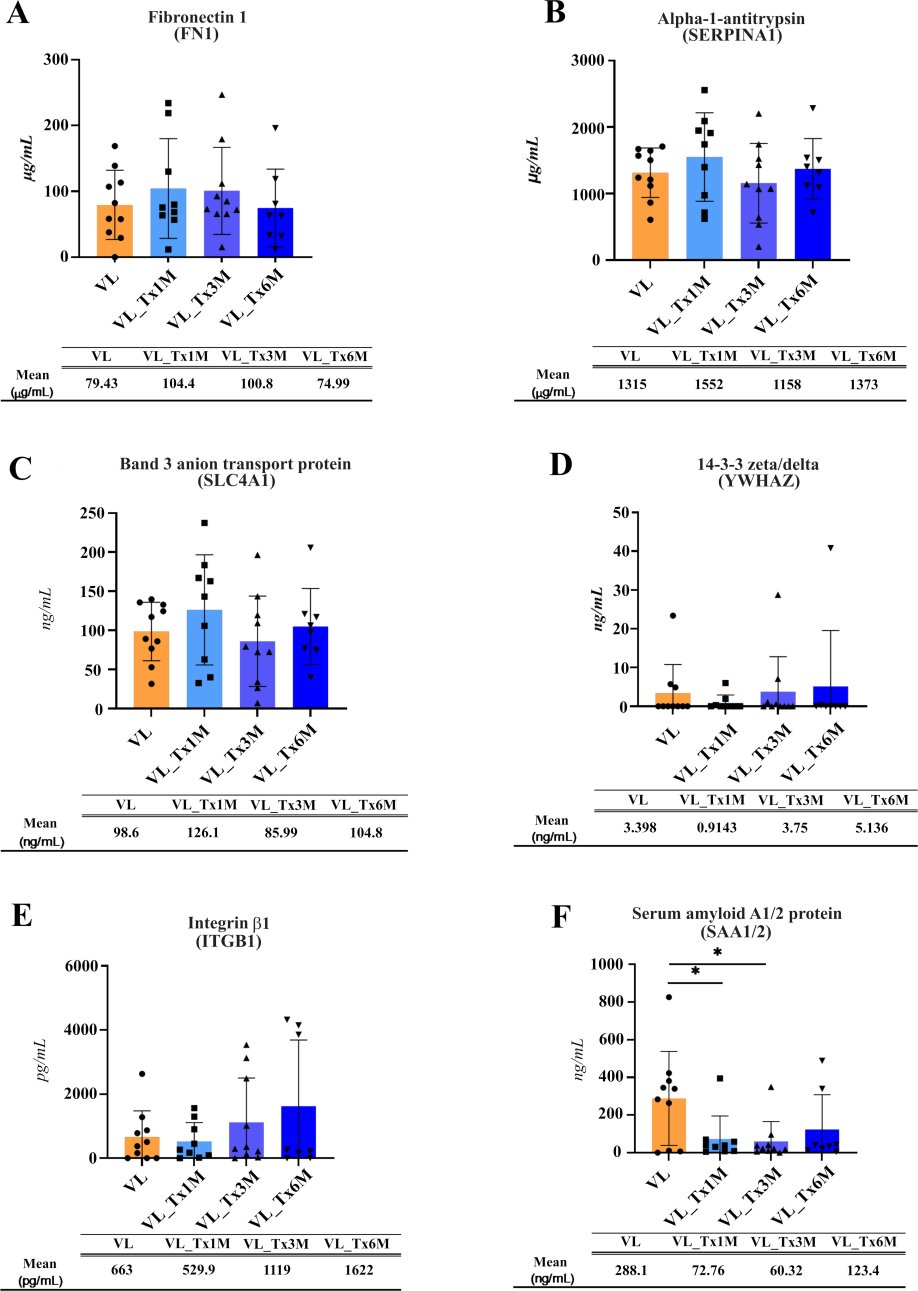
Plasma concentrations of proteins selected as possible biomarkers of cure. The concentration of each selected protein measured via a sandwich ELISA is shown individually for each patient: **(A)** Fibronectin, **(B)** Alpha-1-antitrypsin, **(C)** Band 3 anion transport protein, **(D)** 14-3-3 zeta/deta, **(E)** Integrin β1, **(F)** Serum amyloid A1/2 protein. The mean and standard error for each patient group is also provided. Significant differences are indicated for *p ≤ 0.05. Beneath each figure appears the mean protein concentration recorded for each group.

Plasma expression levels of FN1, SERPINA1, SLC4A1 and YWHAZ concentrations (**Figures 7A–D**) showed no significant differences across the different patient groups.). ITGB1 levels showed a non-significant differing trend (p = 0.633) in plasma samples from patients after 6 months of treatment (mean = 1622 pg/mL) compared to other groups, the most notable difference observed with respect to active VL patients (mean = 663 pg/mL) (**Figure 7E**).

Significant differences were effectively found for SAA1 (**Figure 7F**). SAA1/2 levels were significantly lower in plasma samples from patients at 1 (mean = 72.76 ng/mL) or 3 (mean = 60.32 ng/mL) versus samples from patients with active VL (mean = 288.1 ng/mL; p = 0.0317 and 0.016, respectively). For this protein, ELISA whole plasma and proteomic plasma-derived EV results were consistent, both indicating marked downregulation.

### 3.5 *Leishmania* protein abundances in active VL and treated patients

We also looked for the presence of *L. infantum*-associated proteins in our samples, as identifying parasite proteins is crucial for diagnosis and prognosis and understanding the host-parasite relationship during infection. Peptide sequences were mapped against a protein database derived from the *L. infantum* JPCM5 reference genome (based on v2/2018; http://leish-esp.cbm.uam.es/(50); TriTryDB.org), excluding peptides that also matched the host proteome (human). This analysis identified a total of 46 potential *Leishmania* proteins.

Since only one unique peptide was identified for each protein, we performed additional analyses to ensure the specificity of these proteins as unique to *Leishmania*. To assess potential sequence homologies with proteins from other organisms that could be present in the biological samples, peptides sequences were queried using UniProt BLAST and Peptide search tools, to exclude potential cross-reactivity. This analysis revealed that 19 peptide sequences also showed homology with proteins from other microorganisms, raising doubts about the presence of the corresponding *Leishmania* proteins in the plasma samples (**Supplementary Table 6**). In consequence, we identified 29 *bona fide* proteins associated with the *Leishmania* genome (**Table 3**).

**Table 3:** Identification of *Leishmania bona fide* proteins found in samples from VL patients.

Protein ID	Function	Cellular component	Expression evidence	Reference
LINF_030006400	ATP-binding_cassette_protein_subfamily_F_-_member_1_-_putative	Cytoplasm	General promastigote proteome	(51)
LINF_040009900	Hypothetical_protein_-_conserved	Nucleolus	Purified protein	(52)
LINF_050016600	Hypothetical_protein_-_conserved	–	General promastigote proteome	(51)
LINF_060015800	MYND_finger/SET_domain_containing_protein_-_putative	–	Human plasma EVs	Present study
LINF_070008200	Hypothetical_protein_-_conserved	Axoneme; cytoplasm;cilium	General promastigote proteome	(51)
LINF_070012500	Protein_kinase_-_putative	Cytoplasm	Experimental evidence	(53)
LINF_110007400	Serine/threonine_protein_kinase_-_putative	Cytoplasm	Experimental evidence	(53)
LINF_110008700	Domain_of_unknown_function(DUF2779)_-_putative	Cytoplasm	Human plasma EVs	Present study
LINF_120011000	Fusaric_acid_resistance_protein-like_-_putative	Plasma membrane	Plasma EVs from dogs with leishmaniosis	(26)
LINF_130015300	Subtilisin-like_serine_peptidase	Golgi apparatus; membrane	Human plasma EVs	Present study
LINF_140013300	Hypothetical_protein_-_conserved	Nucleolus	Human plasma EVs	Present study
LINF_140020800	Cilia_and_flagella_associated_protein_44|CFAP44	Cytoplasm; cell projection	General promastigote proteome, flagellar proteome	(54, 55)
LINF_150013800	Kelch_motif/Galactose_oxidase_-_central_domain_containing_protein_-_putative	–	Human plasma EVs	Present study
LINF_180013400	Hypothetical_protein_-_conserved	Cytoplasm; cytosol	General promastigote proteome, metacyclic stage	(51, 54)
LINF_190012900	ATP-binding_cassette_protein_subfamily_F,_member_2|ABCF2	Cytoplamic side of plasma membrane; integral component of membrane	General promastigote proteome, secretome, parasite EVs	(28, 51, 54, 56)
LINF_200007900	Developmentally_regulated_phosphoprotein-like_protein	Mitochondrial matrix	General promastigote proteome	(51)
LINF_220022500	Hypothetical_protein_-_conserved	Cillium;cell projection	Human plasma EVs	Present study
LINF_280023400	Hypothetical_protein_-_conserved	–	Leishmania EVs	(57)
LINF_290006000	C2_domain_in_Dock180_and_Zizimin_proteins_-_putative	Cytoplasm	General promastigote proteome, Leishmania EVs	(28, 54, 57, 58)
LINF_300006600	Leucine-rich_repeat_protein_-_putative	Mitochondrion;cilium	General promastigote proteome	(51, 54)
LINF_300031500	Hypothetical_protein_-_conserved	–	General promastigote proteome	(51)
LINF_310006500	Hypothetical_protein_-_conserved	–	Human plasma EVs	Present study
LINF_310032800	Hypothetical_protein_-_conserved	–	General promastigote proteome, Leishmania EVs	(28, 51, 59–61)
LINF_340038900	Hypothetical_protein_-_conserved	–	Human plasma EVs	Present study
LINF_350036500	Hypothetical_protein_-_conserved	–	Plasma EVs from dogs with leishmaniosis	(26)
LINF_350042600	Hypothetical_protein_-_conserved	–	General promastigote proteome, metacyclic stage proteome, phosphoproteome	(51, 54, 59–61)
LINF_360050100	Glycosyl_hydrolase-like_protein	–	Human plasma EVs	Present study
LINF_360068100	Nitroreductase_family_-_putative	cytoplasm	Leishmania EVs	(28, 57)
LINF_360076500	Hypothetical_protein_-_conserved	–	Human plasma EVs	Present study

The gene ID coding for the protein, its function, and cellular component is indicated as well as evidence for its expression along with previous findings in *Leishmania* studies. A dash in the “Cellular component” column indicates that no specific localization or cellular component has been identified or annotated for the corresponding protein.

Additionally, we conducted an in-depth analysis of the identified *Leishmania* proteins in the literature and the databases TriTrypDB.org, Wikidata, and UniProt to further explore the molecular characteristics and functional annotations of these proteins. Of 46 possible *Leishmania* proteins present in plasma, 36.96% (17 proteins) were classified as ‘hypothetical protein_conserved’ and 41.30% (19 proteins) were classified as ‘putative’, whose functions or biological processes are not yet fully understood. Among the 29 *bona fide* proteins, 14 (48.28%) were classified as ‘hypothetical’ and 10 (34.48%) as ‘putative’. Hypothetical proteins in *Leishmania* constitute a substantial portion of its annotated genome, whose existence is predicted but has not yet been experimentally validated. Their functional annotation is further hindered by the lack of significant sequence homology to proteins of known function. However, their detection in this study confirms their expression and suggests that these proteins may be involved in essential biological processes and represent potential targets for diagnostic and follow-up of VL. Further characterization of these proteins could significantly enhance our understanding of *Leishmania* biology and open new avenues for diagnostic and therapeutic development. For that reason, a literature review was conducted to determine whether these proteins had been previously identified. This review also considered their presence in different parasite forms (amastigotes or promastigotes), their inclusion within the parasite’s secretome or exoproteome, and their description in parasite-derived exosomes. According to the literature and databases, 34 of those 46 proteins have been previously reported. Of these, 26 proteins were described in the *L. infantum* proteome of the Spanish reference *L. infantum* JPCM5 strain, already reported in the different databases and publications (51). Notably, 11 proteins were associated with the secretomes of different species of *Leishmania* (56), and 15 proteins were found in EVs derived from cultured parasites (28, 57, 62). Two proteins were detected in EVs from infected sandfly guts (63), and four were found in EVs from plasma samples from infected dogs (26). Further, two proteins aligned with those described in the exoproteome of *L. infantum* (64), and 14 matched the proteome of *L. donovani* as reported by Adan-Jimenez et al. (54). Importantly, among the 29 *bona fide Leishmania* proteins, 19 have been described in *Leishmania* proteomes while there is no prior evidence for the remaining 10, indicating these could represent novel *Leishmania* proteins experimentally validated in this study and should no longer be considered hypothetical. Detailed descriptions, including molecular functions, peptide sequences, and their literature citings, are provided in **Supplementary Table 6**.

Our analysis of Gene Ontology (GO) terms for the identified proteins served to characterize their potential roles and localization within the parasite (**Supplementary Table 6**). According to the cellular component (CC) terms, most of the identified proteins were localized in the cytoplasm (GO:0005737) or cytosol (GO:0005829), but also associated with membrane structures such as plasma membrane (GO:0005886), Golgi apparatus (GO:0005886), and specific structures within the parasite like axoneme (GO:0005930) and cilium (GO:0005929). In the biological processes (BP) analysis, the proteins identified were related to protein phosphorylation (GO:0006468), intracellular signal transduction (GO:0035556), transmembrane transport (GO:0055085), and ribosomal biogenesis (GO:0042254), reflecting their regulatory roles in metabolism and protein synthesis. Additionally, some proteins are implicated in other processes such as methylation (GO:0042254), redox homeostasis (GO:0045454), and responses to oxidative stress (GO:0034599), suggesting a role in adaptation of the parasite to its environment.

Finally, molecular function (MF) analyses revealed a variety of binding activities, such as ion binding (GO:0043167), ATP binding (GO:0005524), and ribosome binding (GO:0043022), which underscores their importance in energy metabolism and translation. Enzymatic roles include kinase (GO: 0004672), hydrolase (GO:0016787), and oxidoreductase activities (GO:0016491), emphasizing their participation in essential metabolic and signaling pathways.

**Figure 8:**
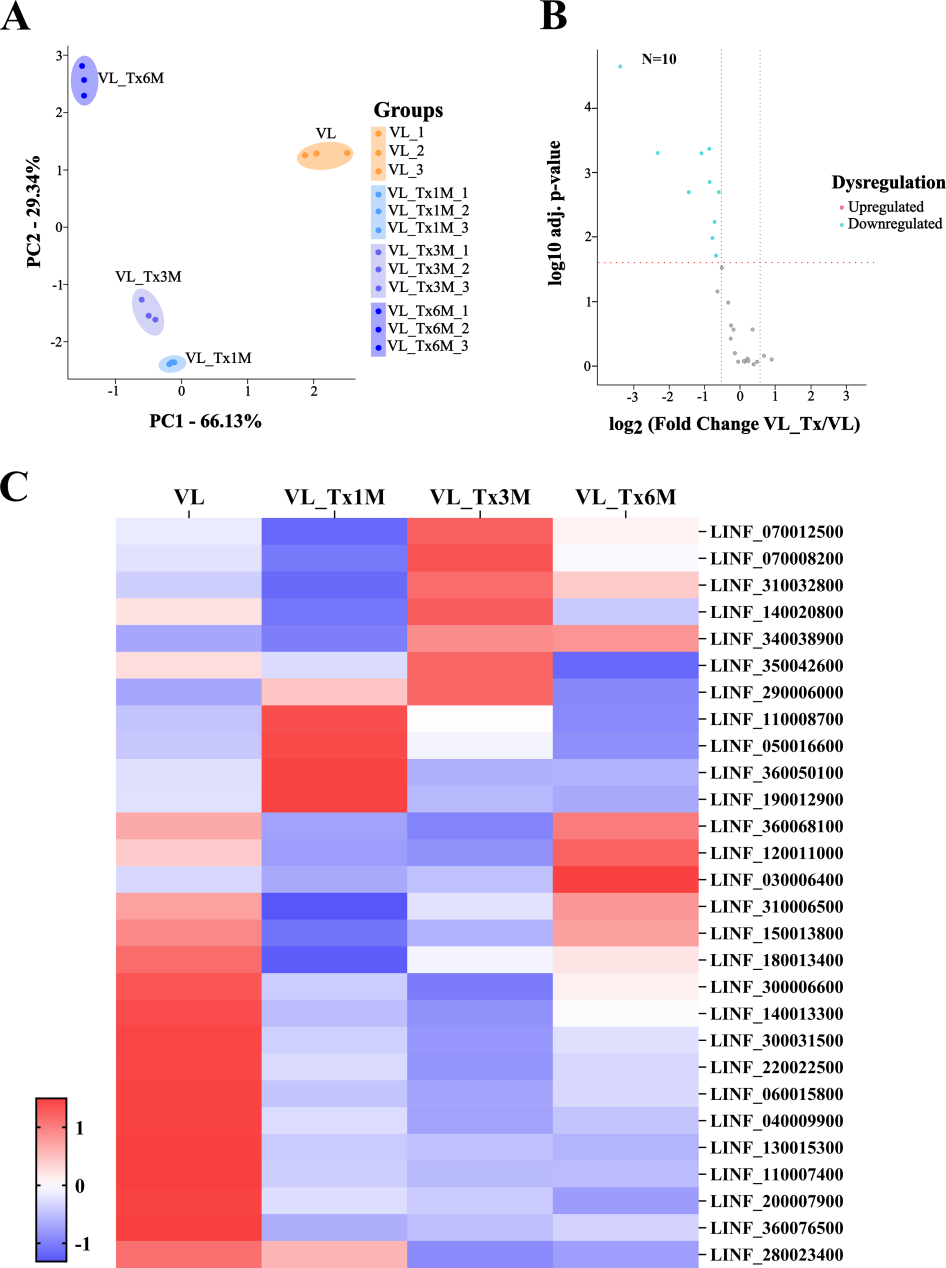
Proteomic profiles of the patient groups VL and VL_Tx-*L. infantum* proteins. **(A)** Variance distributions across different groups (VL, VL_Tx1M, VL_Tx3M, and VL_Tx6M) observed in triplicate samples by PCA showing distinct clustering patterns. **(B)** Volcano plot illustrating differential protein expression in groups VL versus VL_Tx indicating the significant downregulation of 10 proteins (FC≤ 0.67). The two black dashed lines represent a log_2_fold change equal to 0.58 or -0.58. The red dashed line represents a FDR = 0.05. **(C)** Heatmap displaying the proteomic profiles of VL and VL_Tx1M, VL_Tx3M, and VL_Tx6M. Columns correspond to patient groups, while rows represent individual *L. infantum* proteins (LINF), showing their expression patterns across groups.

The 29 selected proteins were subjected to further analysis to evaluate their expression levels across the different patient groups (**Supplementary Table 6**). Proteins whose identified peptides could be also associated with other microorganisms were excluded to avoid introducing noise or generating misleading results. The initial analysis focused on conducting a principal component analysis of the identified proteins. PCA results revealed complete separation of protein expression profiles in active VL patients versus VL_Tx1M, VL_Tx3M and VL_Tx6M, as may be seen in **Figure 8A**. Subsequently, we prepared a volcano plot to compare *Leishmania* protein expression levels between patients with active VL and treated patients (VL_Tx). This analysis revealed that 10 proteins were differentially expressed, all being downregulation in the treated patient compared to the VL group (FC < 0.67) (**Figure 8B**). Finally, we examined the differential expression of these 29 proteins among groups to assess the behavior of their expression following treatment (**Figure 8C**).

The heatmap highlights the expression patterns of 8 of the 10 proteins identified in the volcano plot that showed a FDR < 0.01; these were highly expressed in samples from VL patients. These proteins, LINF_040009900, LINF_060015800, LINF_110007400, LINF_130015300, LINF_140013300, LINF_220022500, LINF_300031500, and LINF_360076500, showed a marked and significant decrease in expression levels in pooled samples after treatment (VL_Tx group). This reduction reflects the therapeutic impact on the disease, suggesting these proteins could be considered biomarkers to monitor disease progression and treatment efficacy.

Interestingly, the heatmap also reveals differences in the abundance of *Leishmania* proteins depending on the time point posttreatment (**Supplementary Table 6**). For instance, 16 proteins showed significant downregulation as early as at 1 month or 3 months posttreatment (**Table 4**). This rapid decline in their expression levels suggests they could serve as early biomarkers of cure.

**Table 4:** Dysregulated *Leishmania* proteins identified in plasma EVs samples from VL patients after treatment compared with VL.

Protein ID	VL_Tx1M	VL_Tx3M	VL_Tx6M
LINF_030006400	–	–	Upregulated
LINF_040009900	Downregulated	Downregulated	Downregulated
LINF_050016600	Upregulated	–	Downregulated
LINF_060015800	Downregulated	Downregulated	Downregulated
LINF_070008200	–	Upregulated	–
LINF_070012500	Downregulated	Upregulated	–
LINF_110007400	–	Downregulated	Downregulated
LINF_110008700	Upregulated	Upregulated	Downregulated
LINF_120011000	Downregulated	Downregulated	–
LINF_130015300	Downregulated	Downregulated	Downregulated
LINF_140013300	Downregulated	Downregulated	Downregulated
LINF_140020800	Downregulated	–	–
LINF_150013800	Downregulated	Downregulated	–
LINF_180013400	Downregulated	–	–
LINF_190012900	Upregulated	–	–
LINF_200007900	Downregulated	Downregulated	Downregulated
LINF_220022500	Downregulated	Downregulated	Downregulated
LINF_280023400	–	Downregulated	Downregulated
LINF_290006000	–	Upregulated	–
LINF_300006600	–	–	–
LINF_300031500	Downregulated	Downregulated	Downregulated
LINF_310006500	–	–	–
LINF_310032800	Downregulated	Upregulated	Upregulated
LINF_340038900	–	–	–
LINF_350036500	Upregulated	–	–
LINF_350042600	–	–	–
LINF_360050100	Upregulated	Downregulated	Downregulated
LINF_360068100	–	–	–
LINF_360076500	–	Downregulated	–

Columns represent relative abundances of the 29 *bona fide* proteins detected in the VL, and VL-Tx1M, VL-Tx3M, and VL-Tx6M groups, comparing samples from treated patients to those with VL. Proteins were considered upregulated (FC≥ 1.5) or downregulated (FC≤ 0.05) only when FDR≤ 0.01. Dashes indicate no detectable dysregulation.

Intriguingly, some of the *Leishmania* proteins featured higher abundances at specific posttreatment time points compared to levels observed during active disease. For example, five proteins LINF_050016600, LINF_110008700, LINF_190012900, LINF_350036500, and LINF_360050100 showed an increased abundance 1 month after treatment. Similarly, in the 3-month posttreatment group, a distinct set of five proteins LINF_070008200, LINF_070012500, LINF_110008700 (which was already elevated in VL_Tx1M), LINF_290006000, and LINF_310032800 were overexpressed. Even 6 months after treatment, LINF_030006400 and LINF_310032800 (the latter was also elevated from VL_Tx3M) were more abundant than in the VL patients.

## 4 Discussion

There is an urgent need for biomarkers of VL able to predict the course of this disease and guide therapeutic decisions. To explore new prognostic indicators, we here conducted a comprehensive analysis of the proteomic profile of the contents of EVs derived from plasma samples of VL patients at different stages, from an active state of disease to different posttreatment time points. Although some proteomic studies have tried to identify biomarkers related to disease-alterations in plasma from *L. donovani* patients (65) and in plasma-derived EVs from *L. infantum* infected dogs (26), no previous study has examined the proteomic profile of plasma-derived EVs from patients infected with *L. infantum* after treatment.

### 4.1 Host immune response and pathways affected by treatment with LAmB

After *Leishmania* infection, the innate system develops an initial acute inflammatory response, including the activation of pathways such as ‘inflammatory response and cell migration’, ‘acute-phase response’, or ‘complement cascade’. Some of the proteins involved in these pathways were found to be predominantly expressed in the EVs of our VL patients when compared to the contents of posttreatment samples. This finding is consistent with an intricate immune response to combat systemic invading pathogens. Following the bite of an infected sandfly, cellular activation triggers the release of molecular mediators that facilitate leukocyte recruitment to the site of *Leishmania* infection. This activation promotes key intracellular signaling pathways, including those regulated by mitogen-activated protein kinase (MAPK), nuclear factor kappa-B (NF-κB), and Janus kinase (JAK)/signal transducer and activator of transcription (STAT) (66). In plasma EVs from patients at 1- and 3 months posttreatment, we observed upregulation of the pathways ‘MAPK family signalling cascade’, ‘integrin signalling’, and ‘regulation of ERK1 and ERK2 cascade’ when compared to patients with active VL. Activation of these pathways could induce the release of proinflammatory cytokines IL-1, IL-6, TNF, transforming growth factor (TGF), and type I interferons (IFN-I), which stimulate the production of acute phase reactants in the liver, such as serum amyloid A (SAA) and alpha-1 antitrypsin (A1AT) (67). We observed higher expression of these proteins in EVs from patients with active disease compared to treated patients. This increase in acute-phase reactants produced in plasma during VL infection has been reported (65, 68–70) and linked to systemic inflammation mediated by cytokines (71, 72). Specifically, an increase in these reactants has been related to a lack of an appropriate response to treatment in canine leishmaniosis (70, 73). According to the latter authors, this suggests that concentrations of acute phase proteins, such as C-reactive protein, are a useful clinical tool to characterize and manage this disease in dogs. However, this biomarker is neither used in veterinary practice today nor suggested for human VL patients. To assess the potential of SERPINA1 (encoding A1AT) and SAA as easily detectable markers of cure, we quantified their levels in whole plasma via ELISA. Contrarily to SERPINA1 which failed to vary across our study groups, SAA levels showed a significant decline soon after treatment (1 month). Given this protein is a marker of inflammation (74), we propose it should be considered an early molecule for use within a biomarker panel for VL follow-up. Other affected pathways were ‘leukocyte activation, migration and adhesion’, and ‘immunoregulatory interactions between a lymphoid and a non-lymphoid cell’. These processes are associated with changes in cell morphology, actin cytoskeleton reorganization, and cellular dynamics. Some peptides such as cathelicidin antimicrobial peptide (CAMP) (75, 76) and vascular cell adhesion protein 1 (VCAM-1) are involved in these mechanisms (77, 78). In a study examining serum levels of VCAM-1 as a marker of relapse in *L. donovani* VL patients, consistently lower levels of this protein were found in cured patients versus those who experienced relapse (79). In line with this observation, we observed the downregulation of VCAM-1 in EVs from *L. infantum* VL patients at 3 months posttreatment, suggesting the potential use of this early biomarker in patients undergoing treatment for VL. Further work involving ELISA is needed to consider VCAM-1 an early easy-to-detect biomarker of relapse in treated VL patients.

As a survival mechanism, *Leishmania* also modulates the VCAM-1/VLA-4 complex, altering the kinetics of infected-monocyte spreading over fibronectin (FN1) (80, 81). By interacting with this protein, *Leishmania* promastigotes impair the activation of parasite-infected macrophages (82, 83). Upregulated FN1 gene expression has been observed in the lymph nodes of Sudanese VL patients after treatment with sodium stibogluconate, compared to the levels observed prior to treatment (84). Similarly, we observed an increase in FN1 levels as well as in ITGB1 and CD9, and ‘integrin signalling’ upregulation in our patients infected with *L. infantum* successfully treated with LAmB, especially at 1 month posttreatment. In agreement, we also observed upregulation of the pathways ‘cellular adhesion’ and ‘regulation of cellular adhesion’ at this time point, suggesting the early recovery of adhesion mechanisms likely contributing to tissue repair and immune cell trafficking. Changes in the levels of these proteins may be related to the immune system’s attempt to re-establish normal cellular adhesion processes and immune cell motility postinfection (78, 80). Although CD9 has been described to modulate viral and bacterial infections, the role of tetraspanins in the pathogenesis of parasitic infections remains unclear (85). Given the systemic effects of ITGB1 and FN1 and the impacts of *Leishmania* on them, both proteins were measured in whole plasma. Only ITGB1 showed a similar profile to that observed in our EV samples by proteomics whereby elevated levels were detected in treated patients, although differences between groups were not significant. As is widely known, EVs can be enriched in certain proteins whose presence varies in whole plasma.

After treatment, we also observed the progressive upregulation of the pathway ‘cell surface interactions at the vascular wall’ and downregulation of proinflammatory kininogen 1 (KNG1). This protein interacts with plasma kallikrein, which plays a role in vessel patency, increasing blood flow, and has anti-thrombotic/profibrinolytic properties (86, 87). Increased vascular permeability induces the entry of leukocytes into inflammation sites, and the downregulation of KNG1 suggests diminished inflammation posttreatment. According to their lower urine levels in patients recovered from *L. donovani* VL, kininogens have been proposed as biomarkers for the diagnosis of infection (88). However, once again, these markers are currently not used for VL patients and there are no commercial assays available to measure urine biomarkers of leishmaniasis.

Another immune system observation noted here after successful treatment was the downregulation of the pathway ‘neutrophil degranulation, migration, chemotaxis and aggregation’ and the upregulation of olfactomedin 4 (OLFM4). This protein is a negative regulator of the NF-κβ pathway, helping prevent excessive inflammation (89). Consistently, our data indicate that the ‘acute inflammatory response’ is already diminished at 1 month posttreatment, reflecting an early shift towards recovery from leishmaniasis. In addition to neutrophils, other phagocytic cells, including monocytes, dendritic cells and macrophages are also recruited to the site of infection. These cells intensely express and secrete S100A9 to modulate inflammatory processes with the induction of inflammatory cytokines, reactive oxygen species (ROS), and nitric oxide (NO) (90). Elevated S100A9 levels have been observed in the serum of experimental mice models infected with *L. infantum* (91). In our study, we found that S100A9 was notably downregulated in patients 1 month after treatment compared to those with active disease. This is consistent with downregulation of the ‘ROS and RNS production’ pathway, suggesting a reduced oxidative stress response as the immune system shifts from active inflammation to a more regulated posttreatment state.

Posttreatment immune modulation also sets the stage for the adaptive immune system’s activation. Plastin-2 (LCP-1) is a protein involved in T-cell polarization, migration, and T-cell–dependent antibody responses (92). This protein has been found upregulated in *L. infantum* infected dogs (93). In our treated patients, LCP-1 expression was downregulated, as well as the pathways associated with antigen processing and presentation, specifically ‘Class I and II MHC mediated antigen processing and presentation’, ‘FCGR dependent phagocytosis’, and ‘T-cell regulation and TCR signalling’. The downregulation of antigen presentation pathways and LCP-1 expression in treated patients likely reflects a reduced antigen load and immune activation, consistent with parasite elimination through effective treatment.

14-3–3 protein zeta/delta (YWHAZ) also plays an important role in promoting T-cell polarization toward Th1 and Th17 populations (94). Additionally, this protein along with UBC and HSP90A, has been identified as a pivotal modulatory factor influencing the immune response, parasite survival and visceralization pathways during *L. donovani* infection (95). Upon differentiation of Th1 cells, these migrate to the infection site to initiate cytotoxic responses. During T-cell transmigration, there is an accumulation of MYH9 at the rear of the cells, facilitating their passage through the membrane (96, 97). In our treated patients, YWHAZ and MYH9 were found upregulated in EVs, indicating polarization to an effective T response following treatment. Our ELISA assay detected increased levels of YWHAZ in the whole plasma of VL patients compared to the levels found 1 month after treatment. High levels of this protein have also been linked to T cell exhaustion markers, thus resulting in T cell dysfunction (98). Closely linked to immune-mediated mechanisms, VL causes significant hematological complications such as hemolytic anemia and premature red blood cell lysis. Infected individuals can show marked degradation of anion channel protein band 3, also known as SLC4A1 (99). This protein plays an important role in maintaining the stability and integrity of the red cell (100). In canine leishmaniosis, studies have shown reduced membrane fluidity and increased cell rigidity, which leads to a greater likelihood of erythrocyte removal from the bloodstream. This could explain the SLC4A1 deficiency observed in erythrocytes from infected dogs (101). In our treated patients, we observed the increased expression of this protein in EVs. This increase could be attributed to the removal of damaged cells and the resolution of hemolysis. Unfortunately, this increase was not mirrored in whole plasma.

Our proteomics analysis of plasma-derived EVs revealed several pathways affected by *Leishmania* infection related to cell adhesion and migration, inflammation and other immune-mediated mechanisms that could also be related to macrophage activation syndrome (102). We found 7 upregulated (SLC4A1, ITGB1, YWHATZ, MYH9, OLFM4, CD9, and FN1) and 9 downregulated (SERPINA1, SAA1, SAA2, HSP90AB1, LCP1, KNG1, VCAM1, S100A9, and CAMP) proteins in EVs from patients treated with LAmB when compared to the active disease state, even early after completing treatment (one month). Among these, six key proteins (SAA, SERPINA1, YWHAZ, ITGB1, FN1, and SLC4A1) were ELISA-detected in whole plasma. Plasma is preferred over EVs due to its broader representation of circulating proteins, ease of processing, and the non-requirement of complex isolation steps. Further, plasma-based assays offer greater accessibility and adaptability, making them attractive for use in the field.

The variations in the levels of two of these proteins (ITGB1 and SAA1) over time were mostly consistent with the EV proteomics results. Importantly, whole plasma SAA1/2 levels significantly declined after effective treatment, indicating their possible use as early biomarkers in the follow-up of VL patients by ELISA. While the trend in ITGB1 levels over time observed here was non-significant, the behavior of this protein may still be interesting for further investigation. Although the potential of SAA1/2 and ITGB1 as biomarkers has been described in other systemic diseases (21, 103–105), this study highlights their significance specifically in the context of leishmaniasis.

### 4.2 *Leishmania*-derived proteins after successful treatment

Our study also identified a subset of *Leishmania* proteins that could serve as biomarkers for monitoring visceral leishmaniasis (VL) and assess treatment efficacy. While previous research has focused on *in vitro* cultured promastigotes (51, 54), their secretomes (56) or EVs derived from cultured parasites (28, 57, 62), or recovered from sandfly guts (63) or from infected animal models (26), this study is the first to detect *Leishmania*-derived proteins in EVs isolated from human patient VL samples.

The framework provided by Esteves et al. (26), who employed canine plasma for the isolation of EVs, guided our analysis of human samples. Among the *Leishmania* proteins identified by these authors in plasma-derived EVs from healthy and diseased dogs, four, LINF_120011000, LINF_350036500, LINF_140007000, and LINF_160020100, were also detected here. These findings not only confirm the detectability of certain proteins across different hosts but also emphasize the need for further studies designed to elucidate their roles in this disease.

Our expression analysis revealed changes in the abundance of *Leishmania* proteins over time, whereby 16 proteins were significantly downregulated as early as 1- or 3 months posttreatment. This stresses the importance of analyzing proteins in detail, as tracking their expression over time can help identify those associated with a rapid response to treatment. Through this analysis, we identified 16 proteins as potential biomarkers of active disease. In effect, these markers could complement the biomarker panel for monitoring cure. In this regard, the peptides and proteins identified could be used to monitor infection via targeted proteomics. Interestingly, we observed higher abundances of certain *Leishmania* proteins at specific time points after treatment compared to the levels detected during active disease.

The detection of *Leishmania*-derived peptides in EVs in treated patients suggests that mass spectrometry can identify low-abundance proteins beyond PCR sensitivity. This highlights the potential of proteomic approaches in uncovering residual parasitic components and underscores the complexity of interpreting antigen persistence. In *Trypanosoma cruzi* infection, it has been already demonstrated that EVs can carry exoantigens during the asymptomatic chronic phase of the disease (106). This may be the result of proteins from dead parasites persisting in plasma EVs, or they could be derived from EVs actively secreted by *Leishmania* during infection that continue to circulate in the bloodstream. Alternatively, they could arise from surviving parasites in visceral tissues that modulate concomitant immunity (107) Notably, 29 of the 33 proteins identified in other studies were found in secretomes or EVs from cultured parasites, or in infected animal models. Among the 10 analyzed proteins featuring a higher abundance pre-treatment than after treatment, 4 had been previously detected in these extracellular components (26, 28, 56, 57, 62). These findings raise concerns about the true origins of *Leishmania* proteins that need to be addressed in future studies.

### 4.3 Study limitations

Our study has some limitations. One limitation is the relatively small sample size and the exclusive use of *L. infantum* samples, which may limit the generalizability to other populations infected with other species of *Leishmania*. Another limitation is the use of pooled samples prior to SC and UC for EV enrichment, which could introduce bias due to potential protein aggregates; nonetheless, PCA analysis supports this approach by revealing distinct protein profiles at VL time points. Additionally, we could not afford the study of all key dysregulated proteins discovered in this study via ELISA in plasma samples. Other candidates could also enrich the suggested panel. In addition, our findings need validation in larger populations. Lastly, the use of plasma instead of EVs for validation could impact the specificity of the detected biomarkers, possibly influencing their diagnostic and prognostic accuracy.

### 4.4 Conclusions and translational impact

In conclusion, the purpose of this study was to compare the proteome of plasma derived EVs during active stage VL and following successful treatment. In addition to a broad set of dysregulated proteins in these vesicles, we discovered several potential markers that could help monitor treatment efficacy by ELISA. Pending confirmation and validation of our results in larger cohorts, we speculate that this approach could be an important new tool to assess the response to therapy. Additionally, detection of *Leishmania* derived proteins in extracellular vesicles provides new insights into infection dynamics and potential biomarkers for future field adapted diagnostic approaches. Our results also provide direction for future studies designed to characterize essential proteins with a role in the pathogenesis of VL or other systemic diseases.

## Data Availability

The mass spectrometry proteomics data have been deposited at the ProteomeXchange Consortium via the PRIDE partner repository with the dataset identifier PXD060604.
