# Supplementary material for: Proteomics of plasma-derived extracellular vesicles from human patients identifies biomarkers for monitoring visceral leishmaniasis therapy
Source: Front Immunol. 2025 Sep 12;16:1646335. doi: 10.3389/fimmu.2025.1646335 (PMC12464055; doi:10.3389/fimmu.2025.1646335)
Supplement: Supplementary file 1 [file Table1.docx]

Supplementary Material

# Supplementary Figures and Tables

## Supplementary Figure 1


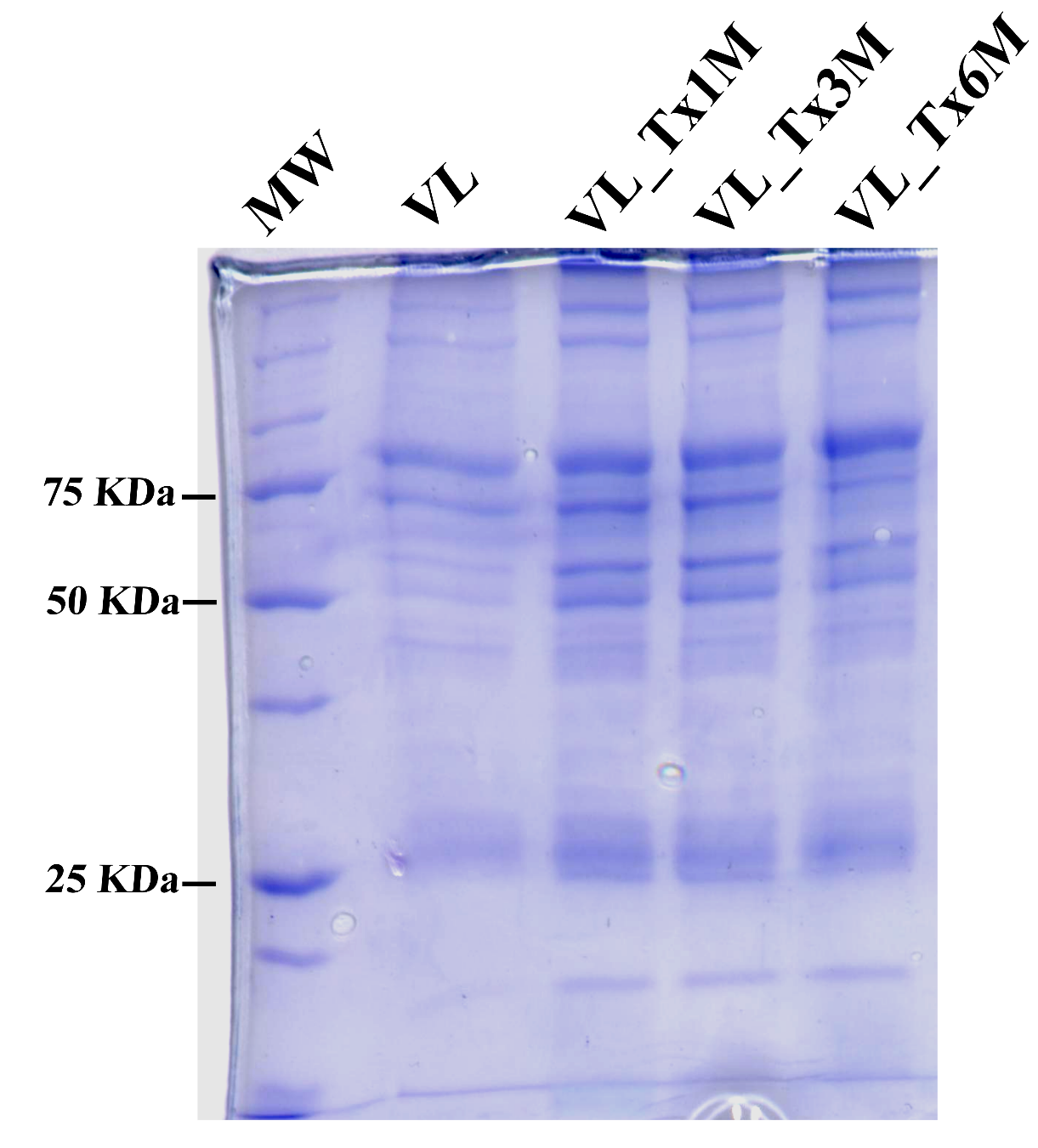


**S1 Fig. SDS-PAGE analysis of protein content in EV preparations.** Representative Coomassie-stained SDS-PAGE gel showing the protein profiles of EVs isolated by SEC+UC from plasma samples of patients at different time points: VL, VL_Tx1M, VL_Tx3M, and VL_Tx6M. MW lane contains molecular weight markers, with key bands indicated at 25 kDa, 50 kDa, and 75 kDa. Each lane contains 10 µg of EV protein.

## Supplementary Figure 2


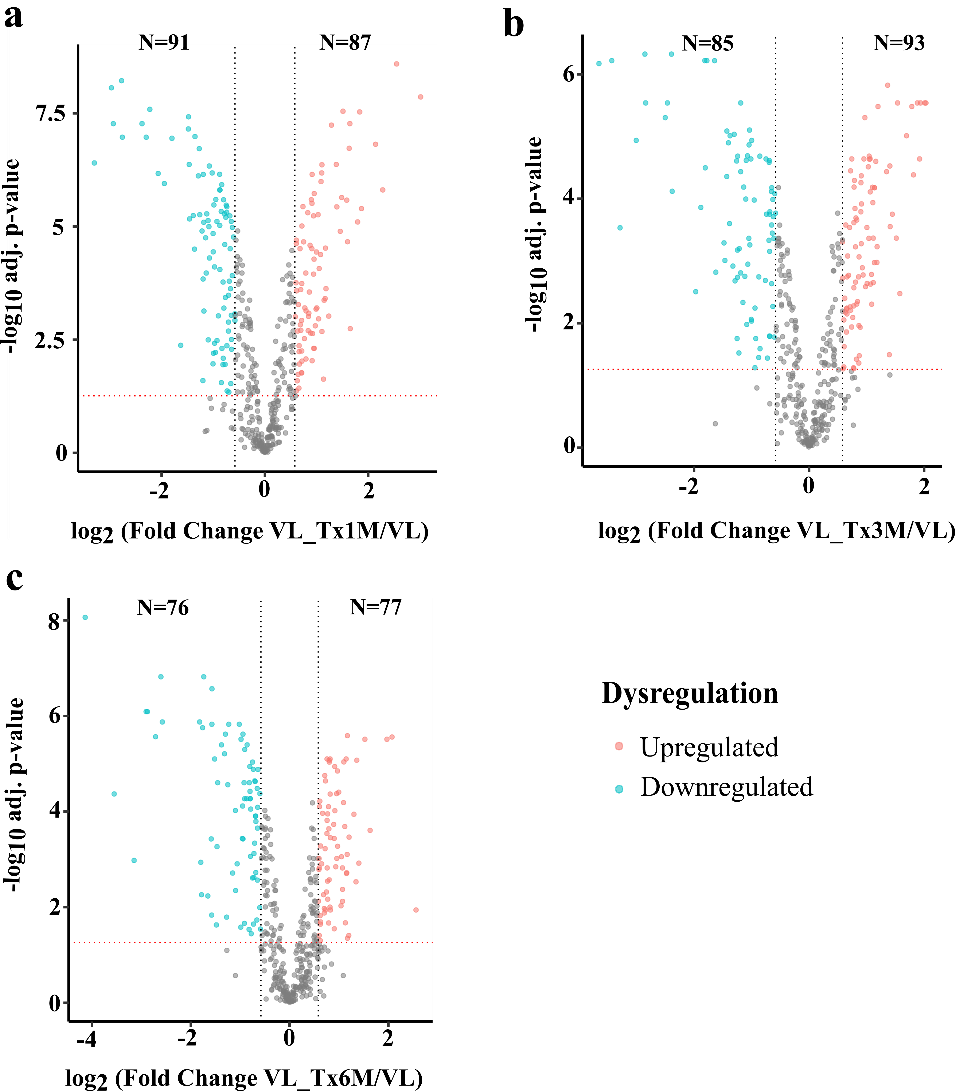


**S2 Fig. Volcano plot illustrating differential protein expression at different timepoints after treatment versus active disease.** a) Tx1M/VL. b) Tx3M/VL and c) Tx6M/VL. The two black dashed lines represent a log2 fold change equal to 0.58 or -0.58. The red dashed line represents an FDR= 0.05.
